# Exposure to deltamethrin leads to gill liver damage, oxidative stress, inflammation, and metabolic disorders of Japanese flounder (*Paralichthys olivaceus*)

**DOI:** 10.3389/ftox.2025.1560192

**Published:** 2025-04-16

**Authors:** Bingbu Li, Guixing Wang, Xinyu Zheng, Mingyang Liu, Yucong Yang, Yuqin Ren, Yitong Zhang, Yufeng Liu, Zhongwei He, Jiangong Ren, Hailong Wan, Wei Cao, Yufen Wang, Xiaoyan Zhang, Jilun Hou

**Affiliations:** ^1^ State Key Laboratory of Mariculture Biobreeding and Sustainable Goods, Beidaihe Central Experiment Station, Chinese Academy of Fishery Sciences, Qinhuangdao, China; ^2^ Hebei Key Laboratory of the Bohai Sea Fish Germplasm Resources Conservation and Utilization, Beidaihe Central Experiment Station, Chinese Academy of Fishery Sciences, Qinhuangdao, China; ^3^ Bohai Sea Fishery Research Center, Chinese Academy of Fishery Sciences, Qinhuangdao, China; ^4^ Ocean College, Hebei Agricultural University, Qinhuangdao, China

**Keywords:** deltamethrin, gills and liver injury, oxidative stress, immunity and metabolism, *Paralichthys olivaceus*

## Abstract

**Introduction:**

Deltamethrin is a pyrethroid insecticide commonly used to kill animal parasites in aquaculture. However, increasing evidence suggests that deltamethrin affects the health of aquatic animals by causing tissue damage and even death.

**Methods:**

In this study, the damage caused by deltamethrin to the gill and liver tissues, as well as its effects on oxidative stress and immune metabolism, were studied in *Paralichthys olivaceus*.

**Results:**

We observed a positive correlation between the residual levels of deltamethrin (Del) and the exposure concentrations, with the highest residue detected in the 0.28 μg/L concentration group (0.0684 mg/kg at 7 days). Then, we observed different degrees of damage to the gill and liver tissues of the Paralichthys olivaceus, including swelling, apical fusion, shedding of gill secondary lamellae, liver cell necrosis, and nuclear vacuolization, by observing tissue sections. Lysozyme enzyme activity increased, whereas catalase and alkaline phosphatase enzyme activities decreased. The liver transcriptome results of the control and high-concentration (0.28 μg/L) groups showed that there were 697 differentially expressed genes, including 390 upregulated and 307 downregulated genes. These differentially expressed genes were significantly enriched in oxidation-reduction, ferroptosis, steroid biosynthesis, and apoptosis pathways.

**Discussion:**

In summary, we found that deltamethrin induces oxidative stress and metabolic disorders in *P. olivaceus* and leads to inflammation. However, the fish body resists such damage through a complex regulatory network. These experimental results provide a theoretical reference for the safe use of deltamethrin in *P. olivaceus*.

## 1 Introduction

Deltamethrin (Del) is a common pyrethroid insecticide widely used in agriculture, animal husbandry, and aquaculture (Trostanetsky et al., 2023). Initially, Del was commonly used to kill animal ectoparasites because of its strong insecticidal effect; However, the most recent research has demonstrated that Del not only exerts toxic effects on target organisms but may also induce varying degrees of harm to non-target species, exhibiting distinct biodiversity-dependent toxicity characteristics (Shi et al., 2024). Del can cause severe pathological changes in *Pangasius hypophthalmus*, such as liver congestion, hepatocyte rupture and necrosis, decreased glomeruli, and shedding of renal tubular epithelial cells (Reddy et al., 2023). Additionally, it can cause neurological and metabolic disorders, oxidative stress, apoptosis, and immune dysfunction in animals (Guardiola et al., 2014; Ding et al., 2017). With the use of insecticides such as organophosphorus (OP), the use of Del and other pyrethroid insecticides is increasing every year (Amweg et al., 2006). Environmental pesticide residues and their harmful effects on aquatic organisms have received widespread attention from researchers (Arslan et al., 2017). Del can effectively eliminate ectoparasites in fish; however, fish possess a reduced capacity to degrade pyrethroid pesticides compared to birds and mammals (Glickman et al., 1982). Therefore, fish are more sensitive to Del (Guardiola et al., 2014). Del exhibits multifaceted toxic effects on zebrafish, primarily characterized by the inhibition of neuronal and skeletal system differentiation and development during embryogenesis, as well as the induction of systemic inflammatory responses and cardiotoxicity (Wang et al., 2024). Moreover, Del exposure has been shown to cause hepatic degeneration, promote cellular apoptosis, induce oxidative stress, and significantly modulate the transcriptional regulation of genes associated with toxicological responses. (Miao et al., 2023). Similarly, there are multiple negative effects of Del on other fish, people are concerned about its toxicity and residual effects on fish (Li et al., 2022). Long-term exposure to Del can result in inflammatory responses, oxidative stress, DNA damage, and apoptosis in common carp (*Cyprinus carpio* L.) (Arslan et al., 2017). It was shown that 24-h acute exposure to Del can eventuate severe desquamation and necrosis of gill epithelial cells, downregulation of some genes involved in innate immune molecules, complement activation and apoptosis-related molecules, and induced immunotoxicity in gibel carp (*Carassius auratus gibelio*) (Wu et al., 2022). In addition, Del affects fish growth and metabolism, inhibits growth and disease resistance in snakehead fish (*Channa argus*), and affects immunity and metabolism in gilthead seabream (*Sparus aurata* L.) (Kong et al., 2021; Guardiola et al., 2014).

The Japanese flounder (*Paralichthys olivaceus*) is an economically important fish in China, Korea, and Japan (Xu et al., 2022). Because of the high-density intensive culture, the residual bait and metabolites in the pool are not cleaned up in time, which can easily lead to parasitic diseases (Jang et al., 2017). Insecticides such as Del are often sprinkled into ponds to treat parasitic diseases of *P*. *olivaceus*, but high concentrations of insecticides may also cause damage to *P*. *olivaceus* larvae. Therefore, it is necessary to conduct studies to evaluate the toxicity of Del and its molecular regulatory mechanisms in *P*. *olivaceus*.

In the present study, we conducted a Del subacute exposure experiment on *P. olivaceus*. We performed transcriptome sequencing and bioinformatics analysis of the liver of *P*. *olivaceus*, prepared tissue sections to analyze the degree of gill and liver damage, and used an enzyme-linked immunosorbent assay (ELISA) to detect oxidative stress and immune and metabolic enzyme activity. This study provide a theoretical reference for the safe use of Del in *P*. *olivaceus*.

## 2 Materials and methods

### 2.1 Experimental fish

The fish were meio-gynogenetic induced *P*. *olivaceus* cultivated at the Beidaihe Central Experimental Station, Chinese Academy of Fishery Sciences, and 800 healthy and active *P*. *olivaceus* (3 months old, 92.27 ± 19.92 g) were selected and temporarily reared in a cement pond.

### 2.2 Determination of the 96 h LC_50_ for *P*. *olivaceus*

Analytical-grade Del (99% purity, T20754, Innochem, China) and acetone (99% purity, 177170010, Innochem, China) were used. Del powder was dissolved in acetone to form Del stock solution at a concentration of 1 mg/L and stored in a refrigerator at −20°C. Del was adjusted to the required concentrations to determine the 96 h LC_50_.

In total, 300 healthy *P*. *olivaceus* were randomly selected from the cement pond and placed in 30 breeding barrels (300 L) for 2 weeks to adapt to the surrounding water environment and reduce external interference. Nine concentration groups (0, 0.2, 0.4, 0.8, 1.6, 2, 4, 8, and 16 μg/L Del) with three replicates each (10 fish per group) were created. The oxygenation equipment was turned on all day to ensure that the dissolved oxygen in the water was ≥8 mg/L. The water temperature, pH and salinity ware kept at 18°C ± 2 °C, 7 ± 1‰ and 30‰, respectively, and for maintaining a preset level of Del concentration, half of the test solution was changed after 24 h. The status of the fish fry was observed every 8 h, and the number of dead fish were recorded. The 96 h LC_50_ (95% confidence interval) was calculated using the probit method.

### 2.3 Subacute del exposure

A total of 225 healthy *P*. *olivaceus* were selected and placed in 15 breeding barrels (15 fish per barrel) for 2 weeks to adapt to the surrounding water environment and reduce external interference. The experimental fish were fed seawater fish feed (Yuequn ocean, China) twice daily. The lethal minimum concentration was 4 μg/L, and the 96 h LC_50_ was 0.35 μg/L. The subacute stress experiment was divided into four groups (0, 0.07, 0.14, 0.28 μg/L), which were 0%, 20%, 40%, and 80% of the 96 h LC_50_, respectively. Water conditions and daily management refer to Section 2.2.

### 2.4 Tissue sample collection

Sampling was conducted on days 1, 2, 4, and seven of the experiment with nine *P*. *olivaceus* randomly selected from each group. Each group of fish was anesthetized with 100 mg/L MS-222 (Sigma-Aldrich GYT0202813, United States) and weighed. Muscle tissue samples were systematically collected and analyzed to quantify the bioaccumulation potential of Del in fish specimens. The tail arterial blood in each group were collected to test serum enzyme activity. Two portions of gill and liver tissues were taken. One sample was soaked in 4% paraformaldehyde to prepare hematoxylin and eosin (H&E) staining sections; Another tissue sample was frozen in liquid nitrogen and stored at −80°C. On day 7, the livers of the control and 0.28 concentration groups were used for transcriptomic and quantitative real-time analysis (qRT-PCR).

### 2.5 Del bioaccumulation assessment

#### 2.5.1 Deltamethrin extraction

Extraction of deltamethrin from fish tissues was performed according to the method described by Niewiadowska et al. (2010).

Operational procedure: Precisely weigh 5 g of muscle tissue into a 50 mL polypropylene centrifuge tube. Add 15 mL acetonitrile and vortex thoroughly for 2 min. Add 1.5 g NaCl, vortex for 1 min, then centrifuge at 4,000 rpm for 5 min. Transfer the supernatant to a new centrifuge tube. Then, add 10 mL acetonitrile-saturated n-hexane to the extract. Vortex vigorously for 3 min and centrifuge at 4,000 rpm for 5 min. Discard the upper organic layer and transfer the lower aqueous phase to a pear-shaped flask. Evaporate the solution to dryness at 45°C using a rotary evaporator. Reconstitute the residue with 2 mL methanol, then dilute with 4 mL ultrapure water. Vortex for homogeneity. Activate the C_18_ Solid-Phase Extraction (SPE) cartridge with 5 mL methanol followed by 5 mL water. Load the diluted extract onto the column at 1 mL/min. Wash with 5 mL methanol-water (3 : 7), discard effluent. Elute target compounds with 6 mL benzene, collecting eluate. The alumina column is activated with 5 mL of acetonitrile, load the primary eluate onto the column. Perform three sequential elutions with 3 mL benzene each. Combine eluates in a 10 mL centrifuge tube and adjust to 5 mL with benzene. The final extracts were subjected to instrumental analysis using a gas chromatograph equipped with an electron capture detector (GC-ECD) under optimized analytical conditions.

#### 2.5.2 Chromatographic conditions

The analytical procedure was adapted from a previously established method (Mahboob et al., 2015). Deltamethrin residues were quantified using an Agilent 7890 b gas chromatograph (United States) equipped with a^63^Ni electron capture detector. The instrument was operated under controlled environmental conditions at 20.6°C and 54% relative humidity. Chromatographic separation was achieved using an HP-5 capillary column (Agilent, United States) with ultra-high purity (99.999%) nitrogen as the carrier gas. The injector and detector temperatures were maintained at 240°C and 300°C, respectively. Samples were introduced in splitless mode with a 1 μL injection volume. The carrier gas flow rate was set at 2.5 mL/min with a purge flow of 25 mL/min. The temperature program was optimized as follows: initial temperature of 100°C held for 0 min, followed by a ramp to 220°C at 30 °C/min (1 min hold), then to 250°C at 5 °C/min (0 min hold), and finally to 290°C at 20 °C/min (5 min hold). Data acquisition and processing were performed using OpenLab CDS ChemStation Chromatographic Software (Agilent, United States). For quantification, a calibration curve was established using deltamethrin standard solutions at concentrations of 10, 20, 50, 100, and 200 μg/L.

### 2.6 Histological processing

Paraffin-embedded sections were prepared following the method refer to Zheng et al. (2021). Gills and liver tissue were fixed overnight in 4% paraformaldehyde. The fixed tissues were excised, subjected to gradient dehydration in ethanol, and subsequently cleared using a mixture of xylene and anhydrous ethanol in a 1:1 ratio. Gill and liver tissue samples were embedded in paraffin wax blocks utilizing a Leica, E.G.,1150H embedding machine (Leica, Germany). The paraffin blocks were then sectioned into thin slices using a Leica RM2255 microtome (Leica). The sections were subsequently dried, dewaxed, and stained with hematoxylin-eosin. Finally, the stained sections were sealed with neutral gum, observed, and photographed using a LEICA DM4 B microscope (Leica).

### 2.7 Serum enzyme activity

To evaluate oxidative stress, immune- and metabolism-related enzymes in the serum, lysozyme (LZM, A050-1-1), acid phosphatase (ACP, A060-1-1), alkaline phosphatase (ALP/AKP, A059-1-1), and catalase (CAT, A007-1-1) were measured using kits (Jiancheng, China). Briefly, the serum was diluted according to the kit requirements. The LZM (530 nm), ACP (520 nm), ALP/AKP (520 nm), and CAT (405 nm) enzyme activities were measured using a UH5300 spectrophotometer (Techcomp, China) at different wavelengths.

### 2.8 Transcriptome analysis

#### 2.8.1 Total RNA extraction and sequencing

Total RNA was extracted from liver tissues using a Tiangen RNA extraction kit (Tiangen DP431, China), and RNA quality was detected using 1% agarose gel electrophoresis. The absorbance ratios at 260 and 280 nm were determined using a NanoDrop 2000 spectrophotometer (Thermo, United States) to verify the quality of the extracted total RNA. The extracted RNA was divided into two samples. One sample was used for transcriptome sequencing, and the other was temporarily stored at −80°C for qRT-PCR expression analysis. Three replicates were used for each group, and six samples (Con 1, Con 2, Con 3, Del 1, Del 2, and Del 3) were prepared. Con 1, Con 2, and Con three are samples from the control group on day 7, while Del 1, Del 2, and Del three are samples from the treatment group on day 7 (0.28 group). Libraries were constructed according to the NEB general library construction method or the strand-specific library construction method (Parkhomchuk et al., 2009), and sequenced using Illumina NovaSeq Xplus (Illumina, United States).

#### 2.8.2 Analysis of differentially expressed genes (DEGs)

Fastp was used to filter to low-quality reads, and Hisat2 was used to create an index of the reference genome, and to compare processed clean reads with the genome of the *P. olivaceus*. Then assembled transcripts based on comparison results (Pertea et al., 2015), and all genes were analyzed in each sample, and their expression levels calculated. Gene expression in different samples was calculated according to fragments per kilobase per million (FPKM) values (Wang et al., 2020). DESeq2 (Anders and Huber, 2010) software was used to screen DEGs. Gene Ontology (GO) and Kyoto Encyclopedia of Genes and Genomes (KEGG) pathway enrichment analyses were performed on the DEG sets using ClusterProfiler software (Yu et al., 2012).

#### 2.8.3 qRT-PCT validation analysis of DEGs

Gene-specific primers for DEGs and *β*-actin used for qRT-PCR are listed in Table 1. Complementary DNA (cDNA) was synthesized utilizing the isolated high-quality total RNA. Then a q225 Real-Time System (Novogen, China) was used to qRT-PCR, the total reaction system is 10 μL, including 5 μL of TB Green Premium Ex Taq II (Takara, Japan), 0.5 μL of the forward and reverse primers (10 μM), 0.5 μL of cDNA and 3.5 μL of RNase-Free water. The cycling conditions were 92°C for 2 min, 40 cycles at 94 °C for 30 s and 60°C for 20 s, and a final extension at 65°C for 5 s and 95°C for 5 s. The relative expression of mRNA of each gene was calculated using the 2^−ΔΔCt^ method (Livak and Schmittgen, 2001).

**TABLE 1:** Gene qRT-PCR primer information.

Primers	Sequence (5′−3 ′)	Use
*tp53i3*-F	GGA​CCA​GAG​AGT​TTG​CTG​CT	Real-time PCR
*tp53i3*-R	TGC​GTA​TCC​TCC​TCC​ACA​GA	
*sccpdh*-F	GGG​ACC​AGT​TCA​AGG​GTA​CG	Real-time PCR
*sccpdh*-R	TTG​GAG​CCA​ACA​GTA​GGC​AG	
*miox*-F	GAT​CCC​AGA​GGA​GGG​TCT​GT	Real-time PCR
*miox*-R	TCT​TGG​ACC​CAG​GGG​ATC​AT	
*acaca*-F	GAT​GCA​GCT​GGA​GGA​GAA​GG	Real-time PCR
*acaca*-R	GCA​GGC​CTG​ACA​TAC​TTG​GT	
*sc5d*-F	TCG​GAG​CGA​TCA​GCT​ACT​TC	Real-time PCR
*sc5d*-R	GCT​GTA​TCC​TCG​GAC​TTC​GG	
*nsdhl*-F	GAC​GAG​CCG​GTG​AGA​TTC​TG	Real-time PCR
*nsdhl*-R	ACC​GAC​CAC​AGG​TTT​GTA​GC	
*p450 1a1*-F	CGA​TAC​GGC​CAT​GTC​TTC​CA	Real-time PCR
*p450 1a1*-R	TCG​GGT​CTG​CCT​GAA​AAC​TC	
*β-actin-*F	GGA​AAC​CAT​GGA​CTG​AGG​CA	Real-time PCR
*β-actin-*R	GGA​AAT​CGT​GCG​TGA​CAT​TAA​G	

### 2.9 Statistical analyses

The data obtained from the experiments are expressed as mean ± standard error (Mean ± SE). The experimental data were subjected to normality and homogeneity of variance assessments utilizing the Shapiro–Wilk test and Levene’s test, respectively, through the application of SPSS software (version 26.0). A one-way analysis of variance (ANOVA) was employed to assess the significance of differences in enzyme activity and gene expression for identical Del exposure durations in *P. olivaceus*, where *P* < 0.05 was significant.

## 3 Results

**FIGURE 1:**
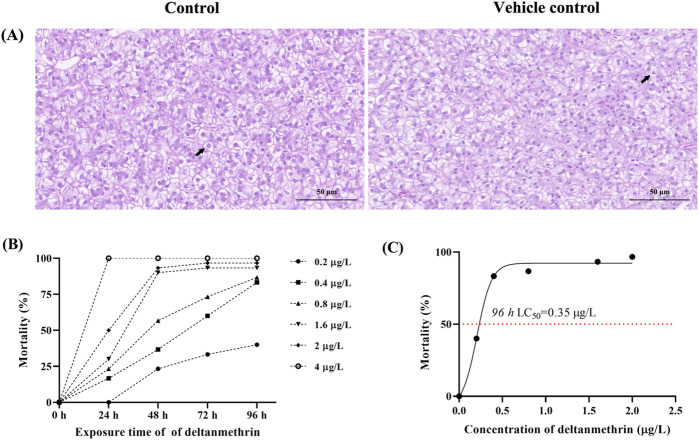
Toxicity of deltamethrin exposure to *Paralichthys olivaceus*. Note: **(A)** Hematoxylin and eosin (H&E) stained sections of liver tissue from control and acetone groups, Black arrows indicate normal hepatocytes; **(B)** Mortality of *Paralichthys olivaceus* at different concentrations of Del; **(C)** 96 h LC_50_ of Del.

The results of the 96 h LC_50_ assay showed that there was no mortality in the control and vehicle control groups, and there was no significant difference in liver tissue structure (Figure 1A), indicating that the solvent had no significant effect on the results of this experiment. Therefore, the control and experimental groups were used in the subsequent analyses.

### 3.1 Determination of the 96 h LC_50_ of del

The status and death of fish at different concentration groups were observed and recorded at 24 h intervals (Figure 1B). The 96 h LC_50_ of *P. olivaceus* Del was calculated to be 0.35 μg/L using SPSS software (Figure 1C). Based on the 96 h LC_50_, we conducted a Del subacute exposure experiment for 7 days with exposure concentrations of 0, 0.07, 0.14, and 0.28 μg/L.

### 3.2 Del residue detection

The quantification of deltamethrin (Del) residues in *P. olivaceus* muscle samples was conducted using the standard curve method, which effectively illustrated the bioaccumulation levels. The standard curve was defined by the equation: *y* = 63.48475*x* + 40.62828, with a high coefficient of determination (*R* ^2^ = 0.99980). Analysis revealed an absence of Del residues in the control group’s muscle samples. A direct correlation was observed between the concentration of Del added and the residue levels detected. Specifically, the Del residue measured 0.0229 mg/kg in the 0.07 μg/L concentration group, 0.0375 mg/kg in the 0.14 μg/L group, and 0.0684 mg/kg in the 0.28 μg/L group.

### 3.3 Histopathological observation

**FIGURE 2:**
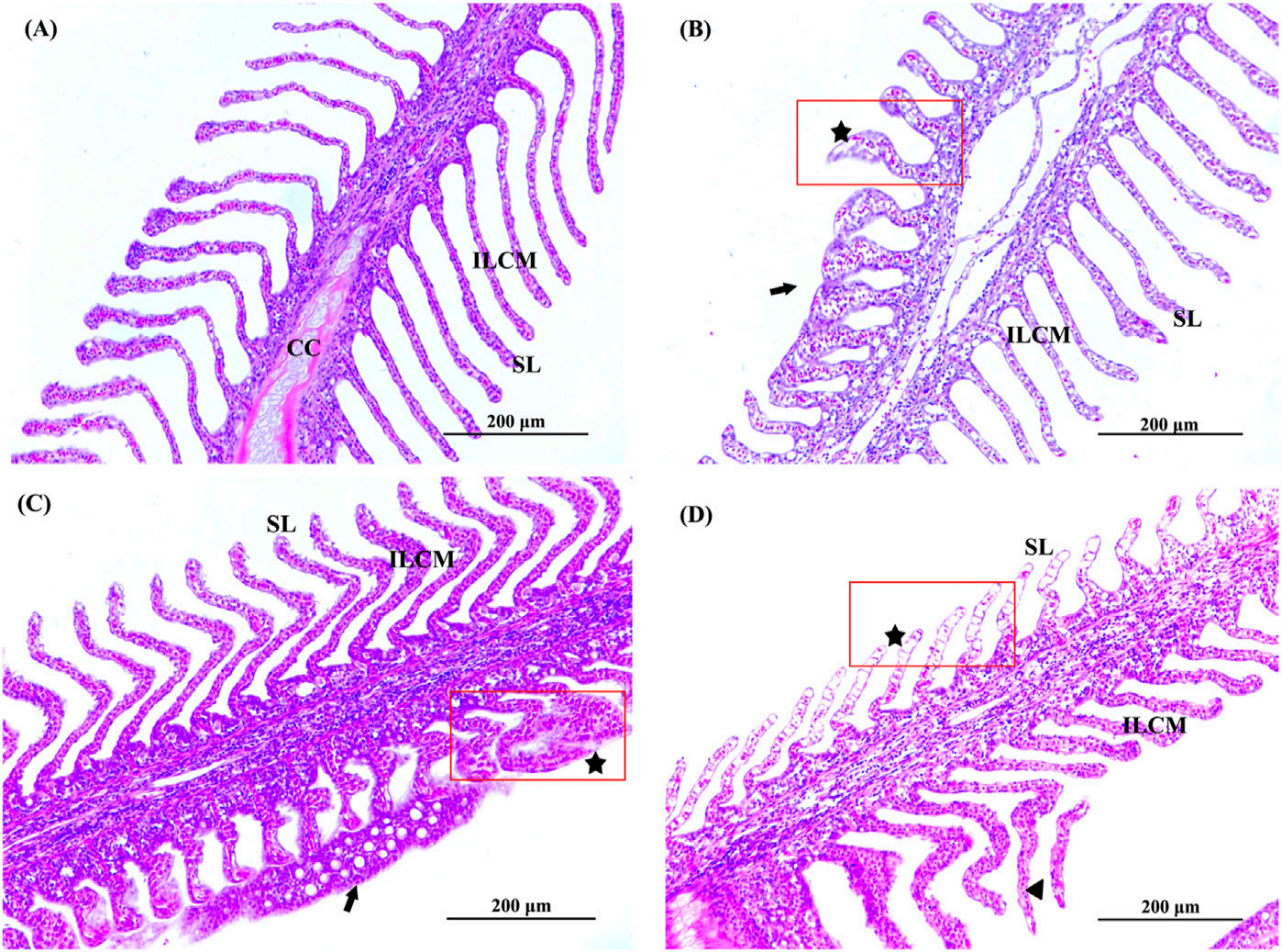
Histopathological observation of gill of Paralichthys olivaceus. Note: **(A)** The control group of gill; **(B)** The 0.07 μg/L group of gill; **(C)** The 0.14 μg/L group of gill; **(D)** The 0.28 μg/L group of gill. Black arrows indicate swelling, fusion, or detachment of secondary lamellae. The black pentagram and red rectangular areas indicate cell necrosis in the secondary lamellae. PF, primary filament; SL, secondary lamellae; ILCM, interlamellar cellular mass; CC, cartilage cell.

**FIGURE 3:**
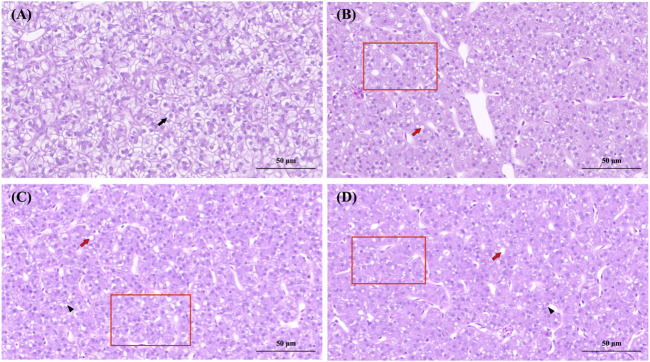
Histopathological observation of the liver of Paralichthys olivaceus. Note: **(A)** The control group of liver; **(B)** The 0.07 μg/L group of liver; **(C)** The 0.14 μg/L group of liver; **(D)** The 0.28 μg/L group of liver. Black arrows indicate normal hepatocytes, red arrows indicate hepatocytes with hepatocyte vacuolization, black triangles indicate hepatocyte necrosis, and red rectangular areas indicate fibrosis due to increased cellular matrix within the liver lobule.

In our experiments, we observed abnormal swimming behaviours, including not lying on the bottom (*P. olivaceus* normally lie on the bottom of the culture bucket), swimming in rapid circles around the bucket, gill cover is not closed and frequent opening of the mouth to swallow seawater. Changes in the histological structure of the gills and liver of *P. olivaceus* were observed after 7 days of Del exposure (Figures 2, 3). Among the gill section observations (Figure 2), the gill tissue of the control group was structurally normal, with symmetrical and secondary lamellae (SL) on both sides of primary filament (PF), cartilage cells (CC) inside primary filament, and a large number of activated cells in interlamellar cellular mass (ILCM) of cells of primary filament and secondary lamellae (Figure 2A). SL showed swelling and apical fusion, and a small number of cells in SL were necrotic in the 0.07 and 0.14 μg/L concentration groups (Figures 2B, C). The epithelial cells of SL were necrotic, the number of erythrocytes was significantly reduced, and there were phenomena such as detachment of SL and degeneration of ILCM in the 0.28 μg/L concentration group (Figure 2D).

Liver lobules in the control group were round and full, with slight nuclear migration (Figure 3A). The nuclei of liver cells in the 0.07 and 0.14 μg/L groups showed vacuolization, fibrosis in some areas of the liver lobule, liver cell congestion, and necrosis in liver cells (Figures 3B, C). The 0.28 μg/L group showed more severe vacuolization of the liver nucleus, more severe liver cell congestion, and extensive fibrosis of the liver lobule accompanied by liver cell lesions and necrosis (Figure 3D).

### 3.4 Serum enzyme activity

**FIGURE 4:**
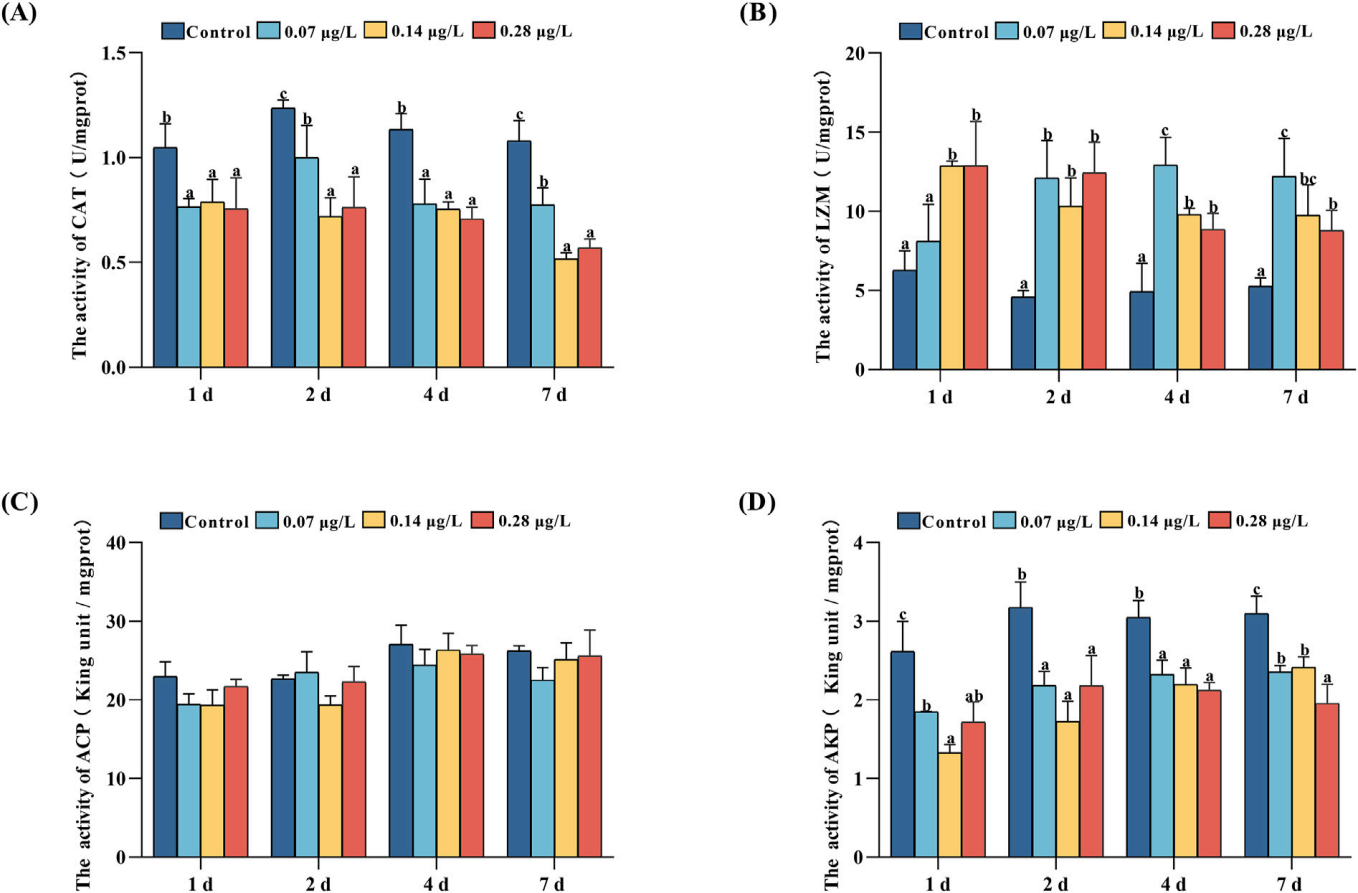
Catalase (CAT), lysozyme (LZM), acid phosphatase (ACP), and alkaline phosphatase (AKP) activities in serum of Paralichthys olivaceus after exposure to different concentrations of Del. Note: **(A)** CAT enzyme activity; **(B)** LZM enzyme activity; **(C)** ACP enzyme activity; **(D)** AKP enzyme activity. Values in the graphs are mean ± SE (n = 3); Different lowercase letters on the way indicate significant differences between different concentration groups at the same time (P < 0.05).

We examined CAT, LZM, ACP, and AKP enzyme activities in the serum of Del-exposed 1, 2, 4, and 7 days *P. olivaceus*, and the results are shown in Figure 4.

The results of CAT enzyme activity (Figure 4A) showed that the control group had the highest CAT enzyme activity from day 1 to day 7 of exposure to Del (*P* < 0.05). Among the treatment groups, the CAT enzyme activity of the 0.07 μg/L group was significantly higher than 0.14 and 0.28 μg/L groups on days 2 and 7.

The results of LZM enzyme activities showed that during the 7-day exposure of different concentrations of Del to *P. olivaceus*, the treatment group had higher LZM activity compared to the control group, except for the low treatment group (0.07 μg/L) on day 1 (Figure 4B), which did not have any significant difference from the control group (*P* > 0.05). Among the treatment groups, the 0.28 μg/L groups had the highest LZM enzyme activity on day 1, and 0.07 μg/L groups had the highest LZM enzyme activity on day 4 and day 7.

The results of AKP enzyme activity showed (Figure 4D) that the AKP activity in the treatment group remained at a lower level compared with the control group (*P* < 0.05). Among the treatment groups, the 0.07 μg/L group had the highest AKP enzyme activity on day 1, the 0.28 μg/L groups had the lowest enzyme activity on day 7.

During the same period, ACP enzyme activity in the treatment group was not significantly different from that in the control group (Figure 4C, *P* > 0.05).

### 3.5 Transcriptome analysis

Six libraries (Con_1, Con_2, Con_3, Del_1, Del_2, Del_3) were constructed by extracting mRNA from liver tissues of the 7 days highest concentration exposure group (0.28 μg/L) and the control group (Con). Sequencing yielded 40,961,900–50,208,994 raw reads with 6.14–7.55G raw bases. After filtering the data, the range of valid reads was 40,817,782–50,208,994, the number of filtered bases was 6.12–7.53G, Q20 was 97.67%–98.88%, Q30 was 94.18%–96.79%, and the GC content was 50.19%–51.33% (Table 2). The data volume was sufficient for subsequent analysis.

**TABLE 2:** Overview of transcriptome sequencing reads and quality filtering of *Paralichthys olivaceus*.

Sample	Raw reads	Raw bases	Clean reads	Clean bases	Q20 (%)	Q30 (%)	GC pct (%)
Con_1	41102690	6.17 G	40972640	6.15 G	98.8	96.6	51.72
Con_2	4,0961900	6.14 G	40817782	6.12 G	98.71	96.42	50.19
Con_3	48188798	7.23 G	48038972	7.21 G	98.8	96.64	51.31
Del_1	48746010	7.31 G	4,3851768	6.58 G	98.72	96.44	51.33
Del_2	46447746	6.97 G	42359732	6.35 G	97.67	94.18	51.6
Del_3	50338014	7.55 G	50208994	7.53 G	98.88	96.79	51.09

**FIGURE 5:**
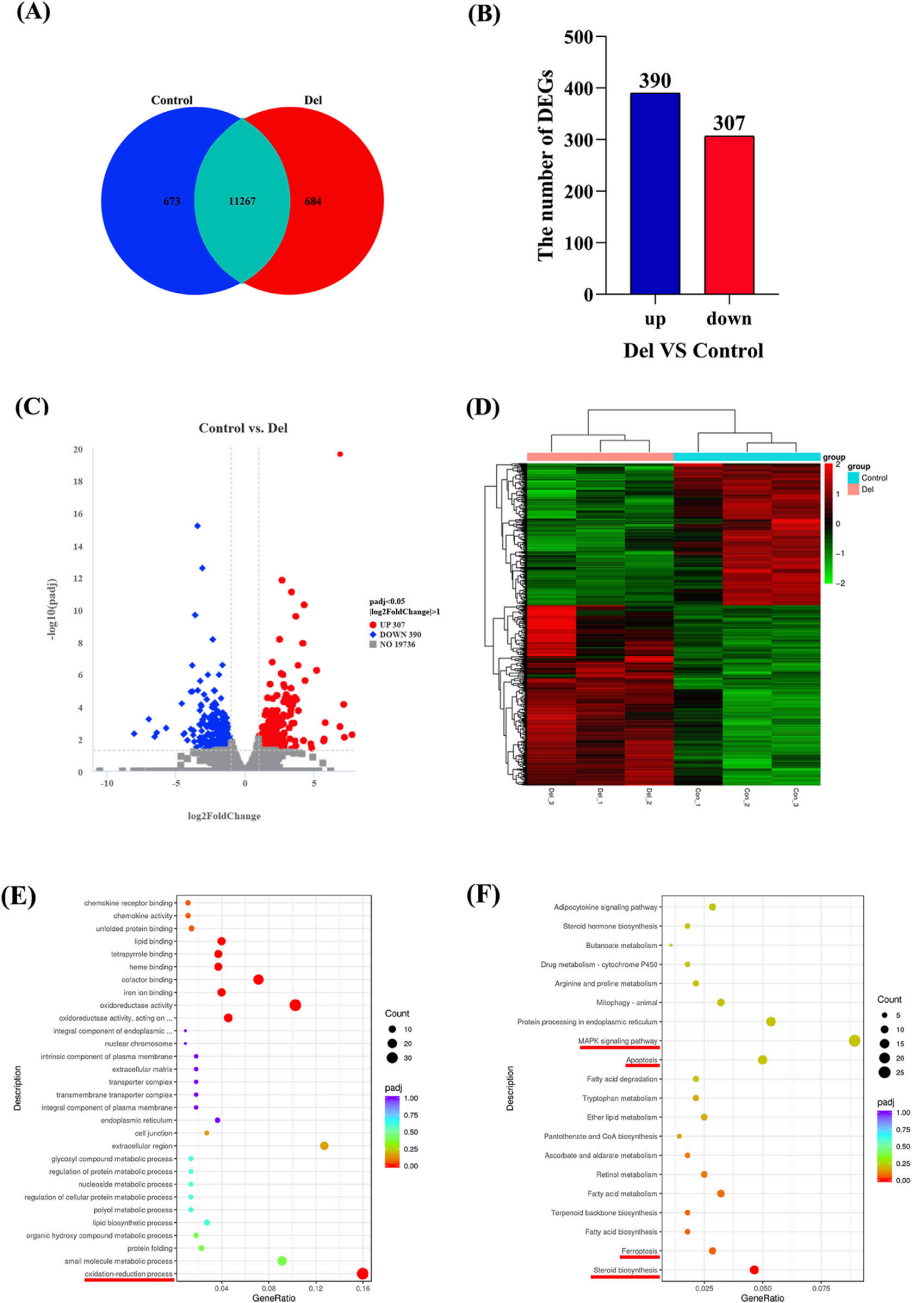
Transcriptome analysis in Control and Del groups in *Paralichthys olivaceus*. Note: **(A)** Co-expression Venn diagram; **(B)** Number of DEGs; **(C)** DEGs volcano map; **(D)** Gene class clustering heat map (*n* = 3); **(E)** Enrichment analysis of Del subacute exposure on liver DEGs GO pathways **(F)** Enrichment analysis of Del subacute exposure on liver DEGs KEGG pathways.

The co-expression Venn diagram results showed (Figure 5A) that there were 11,267 co-expressed genes in the Con and Del groups, and 684 and 673 DEGs, respectively. We used DESeq2 software and set the threshold parameter for differences (*padj* < 0.05, log_2_FoldChange = 1) to analyze the number of differential genes in the control and Del groups. 390 upregulated DEGs and 307 downregulated DEGs were identified (Figures 5B, C). All differentially expressed genes (DEGs) were aggregated into a comprehensive differential gene set. Subsequently, class cluster analysis was conducted on the differential gene sets derived from the six samples within the two groups. The analysis revealed significant differences in the expression patterns between the Con and Del groups (Figure 5D).

### 3.6 GO and KEGG enrichment analysis of DEGs

The results of the GO enrichment analysis showed that 314 signaling pathways were significantly enriched in DEGs when comparing the DEGs of the Con group with those of the Del group (*p* < 0.05), and eight signaling pathways were screened to be significantly enriched in DEGs by correction for multiple hypothesis testing (*padj* < 0.05). These signal pathways were shown oxidation-reduction process (GO:0055114), oxidoreductase activity, acting on paired donors, with incorporation or reduction of molecular oxygen (GO:0016705), oxidoreductase activity (GO:0016491), iron ion binding (GO:0005506), cofactor binding (GO:0048037), heme binding (GO:0020037), tetrapyrrole binding (GO:0046906), and lipid binding (GO:0008289) (Figure 5E). We found that DEGs were significantly enriched in redox- and metabolism-related pathways; and genes such as *sccpdh*, *gys2*, and *hadh* were upregulated, while genes such as *p4501a1*, *miox*, *tp53i3*, *sqlea*, *sc5d*, *nsdhl*, *hmgcr*, and *acaca* were downregulated.

The results of the KEGG enrichment analysis showed that 24 signaling pathways were significantly enriched for DEGs when comparing DEGs in the Con and Del groups (*P* < 0.05). These signaling pathways included ferroptosis (dre04216), fatty acid biosynthesis (dre00061), terpenoid backbone biosynthesis (dre00900), fatty acid metabolism (dre01212), apoptosis (dre04210), and the MAPK signaling pathway (dre04010) (Figure 5F). After correction for multiple hypothesis testing, the steroid biosynthesis (dre01212) pathway was found to be significantly enriched in DEGs (*padj* < 0.05). Notably, the expression of *tnfrsf1a*, *ddit3*, *diabloa*, *gadd45aa*, *il1rap*, *tfr1a*, *lpcat3*, and *ncoa4* was upregulated, whereas the expression of *dhcr7*, *miox*, *sqlea*, *lss*, *dhcr24*, *mvda*, *hmgcr*, *acaca*, and *acsl3* was downregulated in steroid biosynthesis, ferroptosis, fatty acid biosynthesis, terpenoid backbone biosynthesis, apoptosis, and MAPK signaling pathways.

### 3.7 qRT-PCT validation of DEGs

**FIGURE 6:**
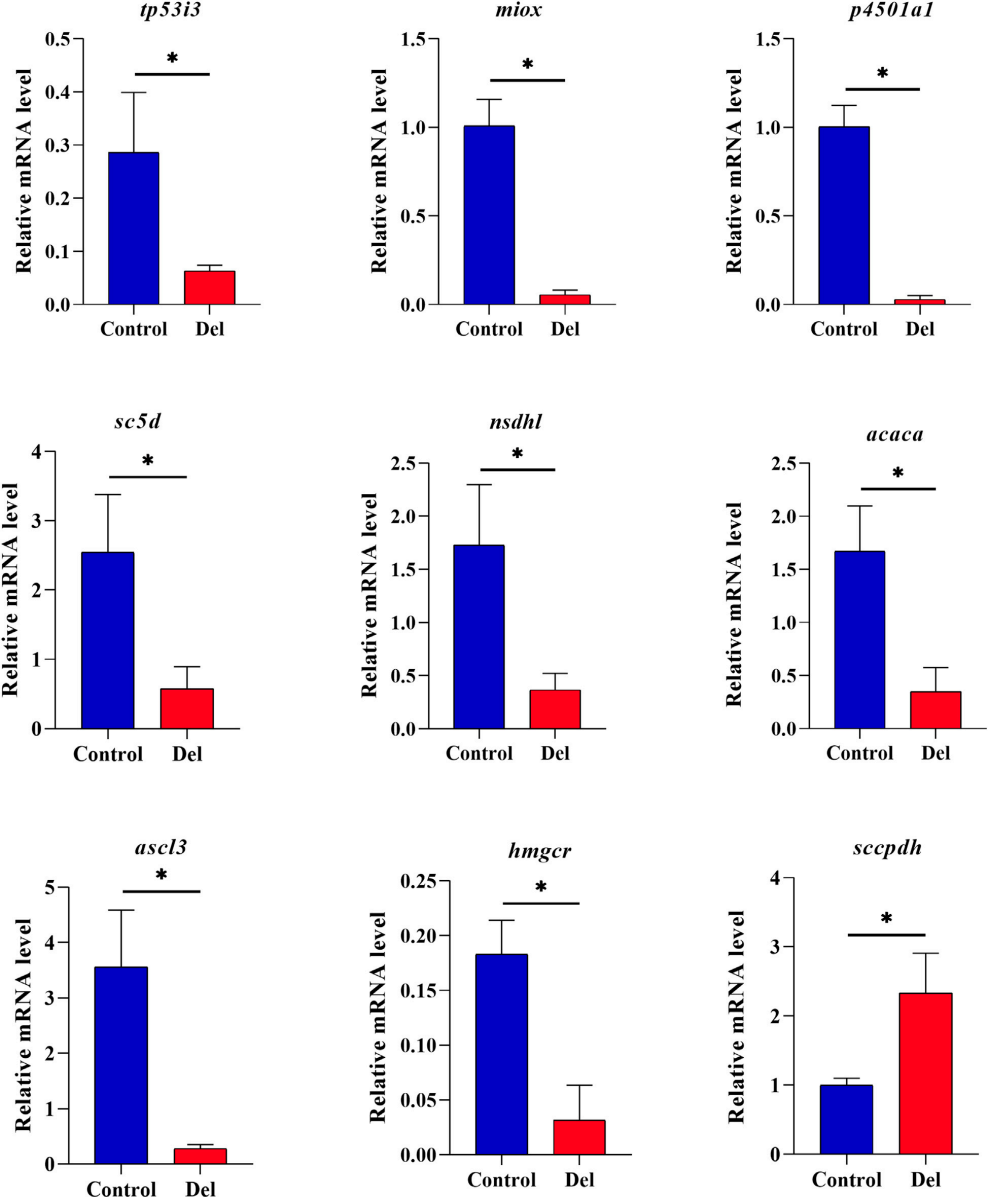
qRT-PCR validation analysis of DEGs in *Paralichthys olivaceus*. Note: Values in the graphs are mean ± SE (*n* = 3); * Indicates that the target gene is significantly different between the control (Control) and treatment (Del) groups.

Nine genes were selected from the oxidation-reduction process, steroid biosynthesis, and ferroptosis pathways and validated by qRT-PCR. The results showed that *tp53i3*, *miox*, *p4501a1*, *sc5d*, *nsdhl*, *hmgcr*, *acaca*, and *acsl3* were downregulated and *hmgcr* was upregulated (Figure 6), which was in agreement with the results of the transcriptome prediction, indicating that the prediction data were real and reliable.

## 4 Discussion

In recent years, an increasing number of studies have shown the negative effects of Del on aquatic animal health (Ning et al., 2020; Alnoaimi et al., 2021). Aquatic animals are more sensitive to pyrethroids, which can also have some negative effects at low doses (Elia et al., 2017).

The half-lethal concentration (LC_50_) is an important parameter for measuring the degree of toxicity of substances present in water to animals (Pereira et al., 2020). Therefore, studying the LC_50_ values of Del in aquatic animals provides a more intuitive understanding of the magnitude of drug toxicity in organisms. Del’s LC_50_ for African clawed frog (*Xenopus laevis*) tadpoles was 6.26 μg/L (168 h); 6.5 μg/L (24 h), 5.0 μg/L (48 h) and 2.8 μg/L (96 h) for *Eriocheir sinensis*; 6.194 μg/L for gibel carp; 1.94 μg/L for snakehead (*C. argus*), and 102.1 μg/L (120 h) for zebrafish embryos (Aydin-Sinan et al., 2012; Ning et al., 2020; Wu et al., 2022; Kong et al., 2021; Miao et al., 2023). In this study, the 96 h LC_50_ of Del was 0.35 μg/L for *P. olivaceus*, indicating that *P. olivaceus* is highly sensitive to Del. Fish, such as *P. olivaceus*, gibel carp, and snakehead, have different tolerances to Del, which may be related to conditions such as species, developmental period, and temperature (Wang et al., 2022; Kadiene et al., 2017).

The study of the bioaccumulation of deltamethrin is an important indicator for analysing its toxicity and detoxification mechanism. In the present study, a positive correlation was observed between the residual levels of deltamethrin (Del) and the exposure concentrations, with the highest residue detected in the 0.28 μg/L concentration group (0.0684 mg/kg at 7 days). Notably, in *Labeo rohita*, exposure to 10 mg/L Del for 96 h resulted in a residual concentration of 2.33 mg/kg (Hazarika et al., 2024). In contrast, the blue mussel (*Mytilus edulis*) demonstrated a relatively low accumulation level (118.3 ng/g at 14 days), with an elimination half-life (t1/2) of 0.83 days (Brooks et al., 2019). Zebrafish exhibited an 8.21% accumulation of deltamethrin after 96 h, while no detectable accumulation was observed in the African clawed frog *(X. laevis*) following 24 h of exposure (Pereira et al., 2023; Wolmarans et al., 2022). These findings highlight that the accumulation of deltamethrin in organisms varies significantly depending on exposure concentration, duration, and species-specific factors.

The gills and liver are integral to the regulation of osmotic pressure and immune metabolism in fish (Cunha et al., 2018). In aqueous environments, Del can directly contact the gill biofilm structures and epithelial cells, destroying the gill tissue structure of fish (Arslan et al., 2017; Wu et al., 2022). In the present study, it was found that the gill tissues of the treatment group showed different degrees of damage (e.g., swelling, fusion, and detachment of SL, and cell necrosis) and were positively correlated with the Del dose, which was similar to the results of gill tissue damage in gibel carp (Wu et al., 2022). Gill morphology is closely related to the tolerance of fish to hypoxic environments (Turko et al., 2020). Therefore, we speculated that chronic exposure to Del causes structural damage to the gill tissues of *P. olivaceus*, inducing oxidative stress in the fish. Del-treated liver tissues showed vacuolization, increased cell gap, dilated central vein of the liver lobule, hepatocyte lesions, and necrosis; and the degree of tissue damage was proportional to the concentration of Del. These liver damage phenomena also appeared in other aquatic organisms, such as gibel carp, Nile tilapia (*Oreochromis niloticus*), marsh frog (*Pelophylax ridibundus*) (Arslan et al., 2017; Kan et al., 2012; Alnoaimi et al., 2021).

Enzymes in the blood are closely related to an animal’s health, metabolism, and immunity, and when the body functions abnormally, the activity of the relevant enzymes in the blood changes rapidly (Gao et al., 2022). The CAT enzyme activities of the treatment groups in this study were all significantly lower than those of the control group (*P* < 0.05), indicating a decrease in the antioxidant capacity of the fish bodies, and the same results were reported in rat gilthead seabream (Xu et al., 2015; Guardiola et al., 2014). The innate immune system constitutes the primary line of defense in fish, and lysozyme (LZM) is an important defense factor of innate immunity, which is one of the important markers of immunotoxicity in fish that can help the organism to defend itself against external stimuli and respond rapidly (Li and Li, 2021). LZM activities in the high-concentration group (0.14 and 0.28 μg/L) were significantly elevated compared to the control group, potentially attributable to the inflammatory response elicited by prolonged exposure to Del (Arslan et al., 2017). AKP activity was significantly reduced in the treatment group compared to the control group (*P* < 0.05), indicating that Del may induce metabolic disturbances (Lu et al., 2019; Guardiola et al., 2014).

Transcriptomics is commonly used to parse molecular regulatory pathways and identify key genes when organisms are subjected to external environmental stress (Wu et al., 2022). The transcriptome results of this study showed that DGEs were significantly enriched in pathways such as ferroptosis, apoptosis, and oxidation-reduction. Genes encoding tumor necrosis factor superfamily members (*tnfrsf1a*) and lysophosphatidylcholine acyltransferase 3 (*lpcat3*) were upregulated. Tnfrsf1a is involved in the immune response and is an important inflammatory factor (Gao et al., 2020). However, overexpression of *lpcat3* ameliorates inflammation and fibrosis (Tian et al., 2024). The upregulation of the *tnfrsf1a* gene in the present study was consistent with the results of LZM enzyme activity in the serum, suggesting that chronic exposure to Del induces an inflammatory response in *P. olivaceus*. The upregulation of *lpcat3* may be caused by self-regulation to alleviate the inflammatory response caused by the upregulation of *tnfrsf1a*. Both gill section and serum CAT enzyme activity demonstrated that the fish were under oxidative stress. Tumor protein p53 Induced protein three gene (*tp53i3*) is among the genes activated by p53, and it plays a critical role in the DNA damage response and apoptosis induced by oxidative stress (Chaudhry et al., 2021). *tp53i3* promotes the proliferation, migration, and invasion of cancer cells, and the downregulation of this gene promotes apoptosis (Chen et al., 2021). Myo-inositol oxygenase (Miox) is a proximal tubular enzyme, and its overexpression exacerbates cellular redox damage and inflammatory responses in acute kidney injury (AKI), and *p53* activation enhances *miox* expression (Deng et al., 2019; Dutta et al., 2017). In this study, both *tp53i3* and *miox* genes were enriched in the oxidation-reduction process pathway, and gene expression was downregulated compared to the control group, which may be the body’s response to defend against oxidative stress by mediating the expression of *tp53i3* and regulating the expression of *p53* and *miox* to attenuate cellular redox damage and inflammatory responses.

In summary, Del causes damage to the gill and liver tissue of *P. olivaceus*. It is worth noting that Del not only causes oxidative stress and metabolic disorders, but also induces inflammatory reactions. We speculated that fish respond to Del stress through a complex regulatory network to counteract the damage caused by oxidative stress and inflammation.

## 5 Limitations

It is noteworthy that deltamethrin has been confirmed to exert multiple toxic effects on fish. This study was conducted exclusively on *P. olivaceus*, and thus, the findings may not be generalizable to other fish species. The experimental parameters, such as exposure concentration and duration, may not fully replicate conditions found in natural ecosystems. Furthermore, the mechanisms underlying the accumulation and metabolic pathways of deltamethrin in *P. olivaceus* remain to be fully elucidated. In future research, we plan to conduct a 14-day subacute exposure experiment to assess the impact of deltamethrin on *P. olivaceus*. This study will involve quantifying its bioaccumulation levels in the fish and determining the corresponding half-life. Subsequently, metabolomic approaches will be employed to delineate the metabolic pathways involved. Lastly, we will explore the potential of phytoremediation strategies, such as the use of *Egeria densa* plants, to mitigate the accumulation of deltamethrin in *P. olivaceus* tissues.

## Data Availability

The original contributions presented in the study are publicly available. This data can be found here: https://bigd.big.ac.cn/gsa/browse/CRA022848.

## References

[B1] Alnoaimi F. Dane H. Sisman T. (2021). Histopathologic and genotoxic effects of deltamethrin on marsh frog, *Pelophylax ridibundus*(Anura: ranidae). Environ. Sci. Pollut. R. 28 (3), 3331–3343. 10.1007/s11356-020-10711-5 32914306

[B2] Amweg E. L. Weston D. P. You J. Lydy M. J. (2006). Pyrethroid insecticides and sediment toxicity in urban creeks from California and Tennessee. Environ. Sci. Technol. 40 (5), 1700–1706. 10.1021/es051407c 16568790

[B3] Anders S. Huber W. (2010). Differential expression analysis for sequence count data. Genome. Biol. 11 (10), r106. 10.1186/gb-2010-11-10-r106 20979621 PMC3218662

[B4] Arslan H. Altun S. Altun S. Özdemir S. (2017). Acute toxication of deltamethrin results in activation of iNOS, 8-OHdG and up-regulation of caspase 3, iNOS gene expression in common carp (*Cyprinus carpio* L.). Aquat. Toxicol. 187, 90–99. 10.1016/j.aquatox.2017.03.014 28399480

[B5] Aydin-Sinan H. Güngördü A. Ozmen M. (2012). Toxic effects of deltamethrin and λ-cyhalothrin on *Xenopus laevis* tadpoles. J. Environ. Sci. Heal. B 47 (5), 397–402. 10.1080/03601234.2012.648545 22424064

[B6] Brooks S. J. Ruus A. Rundberget J. T. Kringstad A. Lillicrap A. (2019). Bioaccumulation of selected veterinary medicinal products (VMPs) in the blue mussel (*Mytilus edulis*). Sci. Total Environ. 655, 1409–1419. 10.1016/j.scitotenv.2018.11.212 30577132

[B7] Chaudhry S. R. Lopes J. Levin N. K. Kalpage H. Tainsky M. A. (2021). Germline mutations in apoptosis pathway genes in ovarian cancer; the functional role of a TP53I3 (PIG3) variant in ROS production and DNA repair. Cell. death. Discov. 7 (1), 62. 10.1038/s41420-021-00442-y 33782397 PMC8007802

[B8] Chen X. J. Zhang W. X. Xu X. Z. (2021). Cyanidin-3-glucoside suppresses the progression of lung adenocarcinoma by downregulating TP53I3 and inhibiting PI3K/AKT/mTOR pathway. World. J. Surg. Oncol. 19 (1), 232. 10.1186/s12957-021-02339-7 34362378 PMC8348822

[B9] Cunha F. D. Sousa N. D. Santos R. F. B. Meneses J. O. Do Couto M. V. S. De Almeida F. T. C. (2018). Deltamethrin-induced nuclear erythrocyte alteration and damage to the gills and liver of *Colossoma macropomum* . Environ. Sci. Pollut. R. 25 (15), 15102–15110. 10.1007/s11356-018-1622-1 29557044

[B10] Deng F. Sharma I. Dai Y. B. Yang M. Kanwar Y. S. (2019). *Myo*-inositol oxygenase expression profile modulates pathogenic ferroptosis in the renal proximal tubule. J. Clin. Invest. 129 (11), 5033–5049. 10.1172/JCI129903 31437128 PMC6819105

[B11] Ding R. Q. Cao Z. F. Wang Y. H. Gao X. B. Lou H. Y. Zhang C. Y. (2017). The implication of p66shc in oxidative stress induced by deltamethrin. Chem. Biol. Interact. 278, 162–169. 10.1016/j.cbi.2017.10.005 28987327

[B12] Dutta R. K. Kondeti V. K. Sharma I. Chandel N. S. Quaggin S. E. Kanwart Y. S. (2017). Beneficial effects of *myo*-inositol oxygenase deficiency in cisplatin-induced AKI. J. Am. Soc. Nephrol. 28 (5), 1421–1436. 10.1681/ASN.2016070744 27895157 PMC5407728

[B13] Elia A. C. Giorda F. Pacini N. Dörr A. J. M. Scanzio T. Prearo M. (2017). Subacute toxicity effects of deltamethrin on oxidative stress markers in rainbow trout. J. Aquat. Anim. Health. 29 (3), 165–172. 10.1080/08997659.2017.1349006 28792275

[B14] Gao J. Yang D. Y. Sun Z. Y. Niu J. Z. Bao Y. H. Liu S. Z. (2022). Changes in blood metabolic profiles reveal the dietary deficiencies of specific nutrients and physiological status of grazing yaks during the cold season in qinghai Province of China. Metabolites 12 (8), 738. 10.3390/metabo12080738 36005610 PMC9413257

[B15] Gao X. Z. Zhang Z. X. Han G. L. (2020). MiR-29a-3p enhances the viability of rat neuronal cells that injured by oxygen-glucose deprivation/reoxygenation treatment through targeting TNFRSF1A and regulating NF-κB signaling pathway. J. Strok. Cerebrovasc. 29 (11), 105210. 10.1016/j.jstrokecerebrovasdis.2020.105210 33066952

[B16] Glickman A. H. Weitman S. D. Lech J. J. (1982). Differential toxicity of trans-permethrin in rainbow trout and mice: I. Role of biotransformation. Toxicol. Appl. Pharm. 66 (2), 153–161. 10.1016/0041-008X(82)90280-0 7164094

[B17] Guardiola F. A. Gónzalez-Párraga P. Meseguer J. Cuesta A. Esteban M. A. (2014). Modulatory effects of deltamethrin-exposure on the immune status, metabolism and oxidative stress in gilthead seabream (*Sparus aurata* L.). Fish. Shellfish. Immunol. 36 (1), 120–129. 10.1016/j.fsi.2013.10.020 24176818

[B18] Hazarika H. Laskar M. A. Krishnatreyya H. Islam J. Kumar M. Zaman K. (2024). Bioaccumulation of deltamethrin and piperonyl butoxide in *Labeo rohita* fish. Ecotoxicol. Environ. Saf. 284, 116908. 10.1016/j.ecoenv.2024.116908 39260219

[B19] Jang Y. H. Subramanian D. Won S. H. Heo M. S. (2017). Immune response of olive flounder (*Paralichthys olivaceus*) infected with the myxosporean parasite *Kudoa septempunctata* . Fish. Shellfish. Immunol. 67, 172–178. 10.1016/j.fsi.2017.06.019 28602738

[B20] Kadiene E. U. Bialais C. Ouddane B. Hwang J. S. Souissi S. (2017). Differences in lethal response between male and female calanoid copepods and life cycle traits to cadmium toxicity. Ecotoxicology 26 (9), 1227–1239. 10.1007/s10646-017-1848-6 28990129

[B21] Kan Y. Cengiz E. I. Ugurlu P. Yanar M. (2012). The protective role of vitamin E on gill and liver tissue histopathology and micronucleus frequencies in peripheral erythrocytes of *Oreochromis niloticus* exposed to deltamethrin. Environ. Toxicol. Pharmacol. 34 (2), 170–179. 10.1016/j.etap.2012.03.009 22534510

[B22] Kong Y. D. Li M. Shan X. F. Wang G. Q. Han G. H. (2021). Effects of deltamethrin subacute exposure in snakehead fish, *Channa argus*: biochemicals, antioxidants and immune responses. Ecotox. Environ. Safe. 209, 111821. 10.1016/j.ecoenv.2020.111821 33360593

[B23] Li L. C. Liu S. G. Yin Y. Zheng G. M. Zhao C. Ma L. S. (2022). The toxicokinetics and risk assessment of pyrethroids pesticide in tilapia (*Oreochromis mossambicus*) upon short-term water exposure. Ecotox. Environ. Safe. 241, 113751. 10.1016/j.ecoenv.2022.113751 35691199

[B24] Li Z. H. Li P. (2021). Effects of the tributyltin on the blood parameters, immune responses and thyroid hormone system in zebrafish. Environ. Pollut. 268, 115707. 10.1016/j.envpol.2020.115707 33007597

[B25] Livak K. J. Schmittgen T. D. (2001). Analysis of relative gene expression data using real-time quantitative PCR and the 2^-ΔΔCT^ method. Methods 25 (4), 402–408. 10.1006/meth.2001.1262 11846609

[B26] Lu Q. R. Sun Y. Q. Ares I. Anadon A. Martinez M. Martinez-Larranaga M. R. (2019). Deltamethrin toxicity: a review of oxidative stress and metabolism. Environ. Res. 170, 260–281. 10.1016/j.envres.2018.12.045 30599291

[B27] Mahboob S. Niazi F. AlGhanim K. Sultana S. Al-Misned F. Ahmed Z. (2015). Health risks associated with pesticide residues in water, sediments and the muscle tissues of *Catla catla* at Head Balloki on the River Ravi. Environ. Monit. Assess. 187 (3), 81. 10.1007/s10661-015-4285-0 25655128

[B28] Miao W. Y. Jiang Y. M. Hong Q. Y. Sheng H. D. Liu P. P. Huang Y. F. (2023). Systematic evaluation of the toxicological effects of deltamethrin exposure in zebrafish larvae. Environ. Toxicol. Phar. 100, 104155. 10.1016/j.etap.2023.104155 37209891

[B29] Niewiadowska A. Kiljanek T. Semeniuk S. Zmudzki J. (2010). Determination of pyrethroid residues in meat by gas chromatography with electron capture detection. B Vet. I Pulawy 54 (4), 595–599.

[B30] Ning M. X. Hao W. J. Cao C. Xie X. J. Fan W. F. Huang H. (2020). Toxicity of deltamethrin to Eriocheir sinensis and the isolation of a deltamethrin-degrading bacterium, *Paracoccus* sp. P-2. Chemosphere 257, 127162. 10.1016/j.chemosphere.2020.127162 32485514

[B31] Parkhomchuk D. Borodina T. Amstislavskiy V. Banaru M. Hallen L. Krobitsch S. (2009). Transcriptome analysis by strand-specific sequencing of complementary DNA. Nucleic. acids. Res. 37 (18), e123. 10.1093/nar/gkp596 19620212 PMC2764448

[B32] Pereira A. M. Camargo-Mathias M. I. Daemon E. Peconick A. P. Lima-Souza J. R. Oliveira P. R. (2020). Efficacy of carvacrol on *Rhipicephalus* (*Boophilus) microplus* engorged female ticks (Canestrini, 1887) (Acari: ixodidae): effects on mortality and reproduction. Nat. Prod. Res. 34 (23), 3428–3431. 10.1080/14786419.2019.1569657 30761912

[B33] Pereira N. S. Munhoz-Garcia G. V. Takeshita V. Pimpinato R. F. Tornisielo V. L. Mendes K. F. (2023). *Egeria densa* remediates the aquatic environment and reduces 14C-deltamethrin bioaccumulation in *Danio rerio* . J. Environ. Sci. Heal. B 58 (6), 500–505. 10.1080/03601234.2023.2232277 37430469

[B34] Pertea M. Pertea G. M. Antonescu C. M. Chang T. C. Mendell J. T. Salzberg S. L. (2015). StringTie enables improved reconstruction of a transcriptome from RNA-seq reads. Nat. Biotechnol. 33 (3), 290–295. 10.1038/nbt.3122 25690850 PMC4643835

[B35] Reddy C. P. L. Manikandavelu D. Arisekar U. Ahilan B. Uma A. Jayakumar N. (2023). Toxicological effect of endocrine disrupting insecticide (deltamethrin) on enzymatical, haematological and histopathological changes in the freshwater iridescent shark, Pangasius hypothalamus. Environ. Toxicol. Pharmacol. 101, 104201. 10.1016/j.etap.2023.104201 37391053

[B36] Shi T. L. Zhang Q. W. Chen X. Y. Mao G. H. Feng W. W. Yang L. Q. (2024). Overview of deltamethrin residues and toxic effects in the global environment. Environ. Geochem. Health. 46 (8), 271. 10.1007/s10653-024-02043-x 38954040

[B37] Tian Y. Jellinek M. J. Mehta K. Seok S. M. Kuo S. H. Lu W. (2024). Membrane phospholipid remodeling modulates nonalcoholic steatohepatitis progression by regulating mitochondrial homeostasis. Hepatology 79 (4), 882–897. 10.1097/HEP.0000000000000375 36999536 PMC10544743

[B38] Trostanetsky A. Quinn E. Rapaport A. Harush A. Gottlieb D. (2023). Efficacy of deltamethrin emulsifiable concentrate against stored-product insects. J. Stored. Prod. Res. 101, 102072. 10.1016/j.jspr.2022.102072

[B39] Turko A. J. Cisternino B. Wright P. A. (2020). Calcified gill filaments increase respiratory function in fishes. P. Roy. Soc. B-biol. Sci. 287 (1920), 20192796. 10.1098/rspb.2019.2796 PMC7031667 32075528

[B40] Wang J. Li H. Q. Liu Y. Y. Andrzejczyk N. K. Qiao K. Ma Y. F. (2024). Contribution of immune responses to the cardiotoxicity and hepatotoxicity of deltamethrin in early life stage zebrafish (*Danio rerio*). Environ. Sci. Technol. 58 (22), 9515–9524. 10.1021/acs.est.3c10682 38687472

[B41] Wang Y. H. Chen C. Yang G. L. Wang X. Q. Wang Q. Weng H. B. A. (2022). Combined lethal toxicity, biochemical responses, and gene expression variations induced by tebuconazole, bifenthrin and their mixture in zebrafish (*Danio rerio*). Ecotox. Environ. Safe. 230, 113116. 10.1016/j.ecoenv.2021.113116 34979316

[B42] Wang Z. Y. Leushkin E. Liechti A. Ovchinnikova S. Mössinger K. Brüning T. (2020). Transcriptome and translatome co-evolution in mammals. Nature 588 (7839), 642–647. 10.1038/s41586-020-2899-z 33177713 PMC7116861

[B43] Wolmarans N. J. Bervoets L. Meire P. Wepener V. (2022). Sub-lethal exposure to malaria vector control pesticides causes alterations in liver metabolomics and behaviour of the African clawed frog (*Xenopus laevis*). Comp. Biochem. Physiol. C Toxicol. Pharmacol. 215, 109173. 10.1016/j.cbpc.2021.109173 34492387

[B44] Wu H. Gao J. W. Xie M. Xiang J. Zuo Z. L. Tian X. (2022). Histopathology and transcriptome analysis reveals the gills injury and immunotoxicity in gibel carp following acute deltamethrin exposure. Ecotox. Environ. Safe. 234, 113421. 10.1016/j.ecoenv.2022.113421 35304335

[B45] Wu H. Yuan X. P. Gao J. W. Xie M. Tian X. Xiong Z. Z. (2023). Conventional anthelmintic concentration of deltamethrin immersion disorder in the gill immune responses of crucian carp. Toxics 11 (9), 743. 10.3390/toxics11090743 37755753 PMC10534886

[B46] Xu M. Y. Wang P. Sun Y. J. Wang H. P. Liang Y. J. Zhu L. (2015). Redox status in liver of rats following subchronic exposure to the combination of low dose dichlorvos and deltamethrin. Pestic. Biochem. Physiol. 124, 60–65. 10.1016/j.pestbp.2015.04.005 26453231

[B47] Xu X. W. Zheng W. W. Yang Y. M. Hou J. L. Chen S. L. (2022). High-quality Japanese flounder genome aids in identifying stress-related genes using gene coexpression network. Sci. Data. 9 (1), 705. 10.1038/s41597-022-01821-5 36385241 PMC9668919

[B48] Yang C. Lim W. Song G. (2021). Immunotoxicological effects of insecticides in exposed fishes. Comp. Biochem. Phys. C 247, 109064. 10.1016/j.cbpc.2021.109064 33905824

[B49] Yu G. C. Wang L. G. Han Y. Y. He Q. Y. (2012). clusterProfiler: an R Package for comparing biological themes among gene clusters. Omics 16 (5), 284–287. 10.1089/omi.2011.0118 22455463 PMC3339379

[B50] Zheng T. Song Z. Qiang J. Tao Y. F. Zhu H. J. Ma J. L. (2021). Response in hybrid yellow catfish (*Tachysurus fulvidraco*♀ x *P. vachellii*♂) through TLR/NLR signaling pathways and regulation of mucus secretion. Front. Immunol. 12, 741359. 10.3389/fimmu.2021.740359 PMC8545808 34712228

