# Supplementary figures and images for: Exposure to deltamethrin leads to gill liver damage, oxidative stress, inflammation, and metabolic disorders of Japanese flounder (Paralichthys olivaceus)
Source: Front Toxicol. 2025 Apr 16;7:1560192. doi: 10.3389/ftox.2025.1560192 (PMC12041085; doi:10.3389/ftox.2025.1560192)

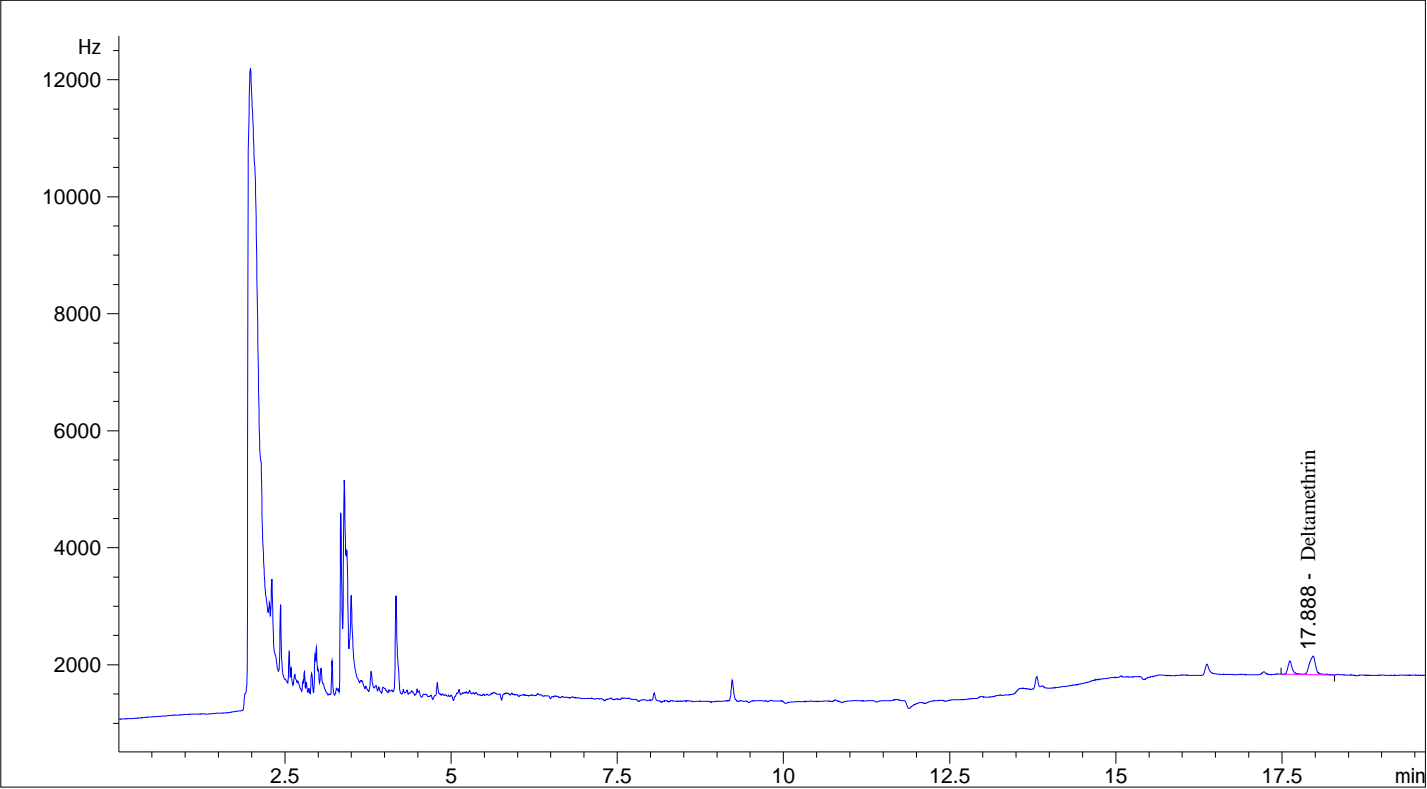

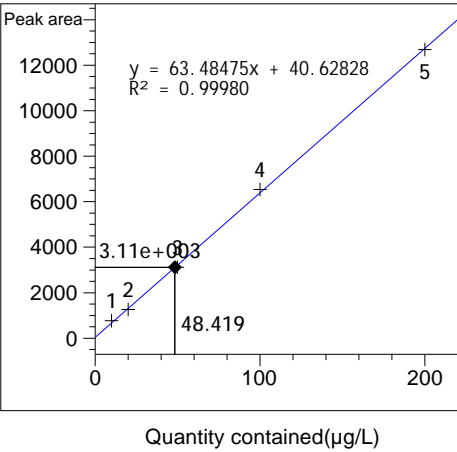

Supplement: Supplementary file 1 [file DataSheet2.pdf]

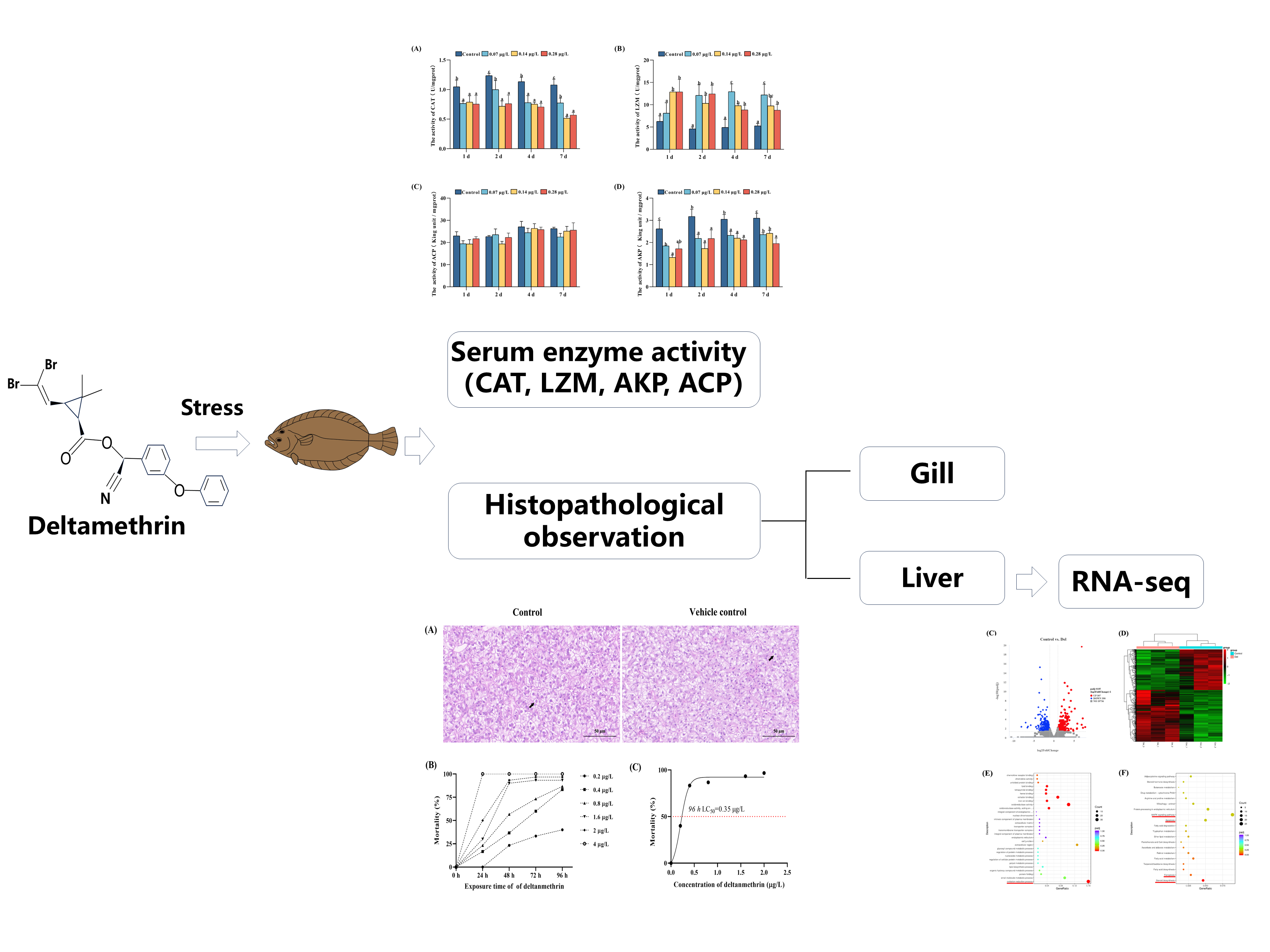

Supplement: Supplementary file 3 [file Image1.TIF]

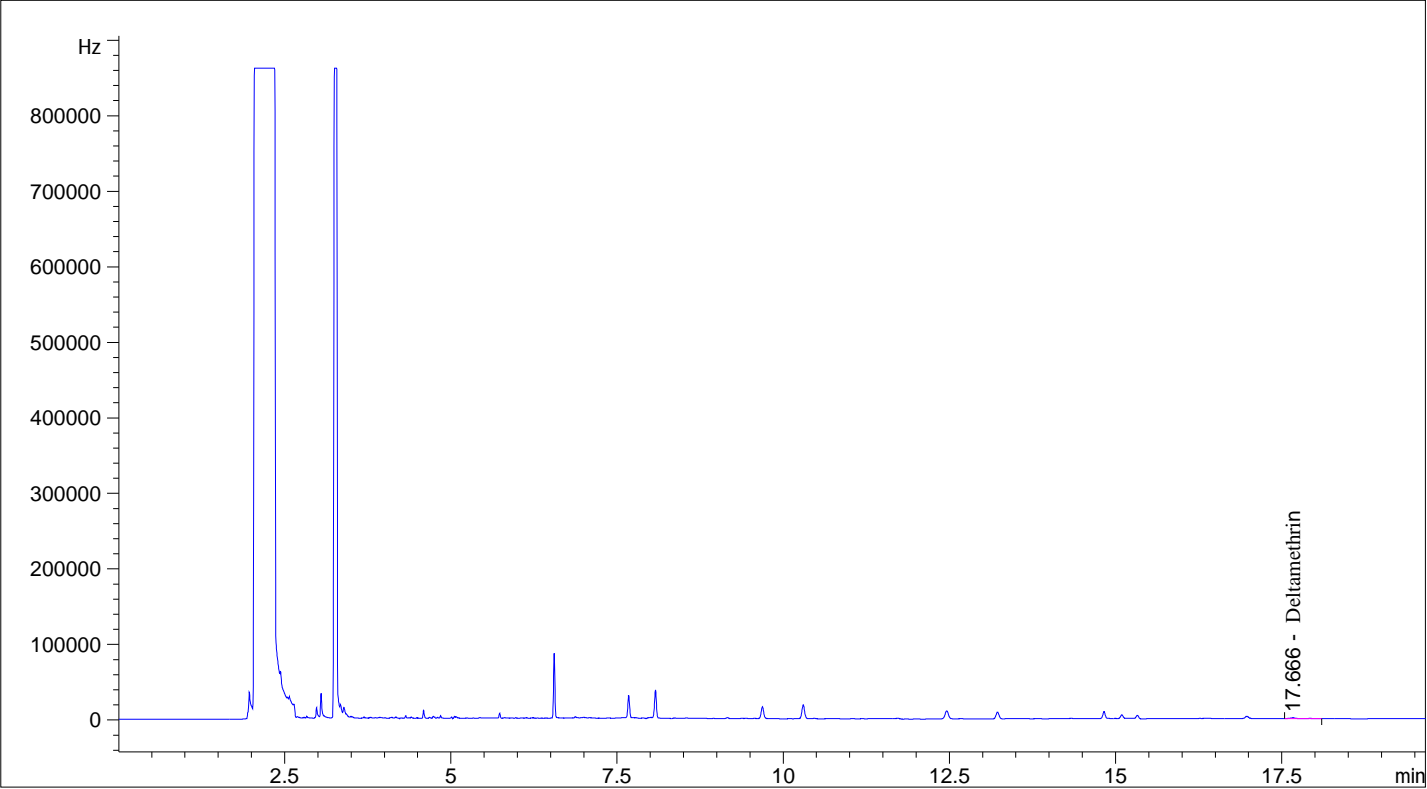

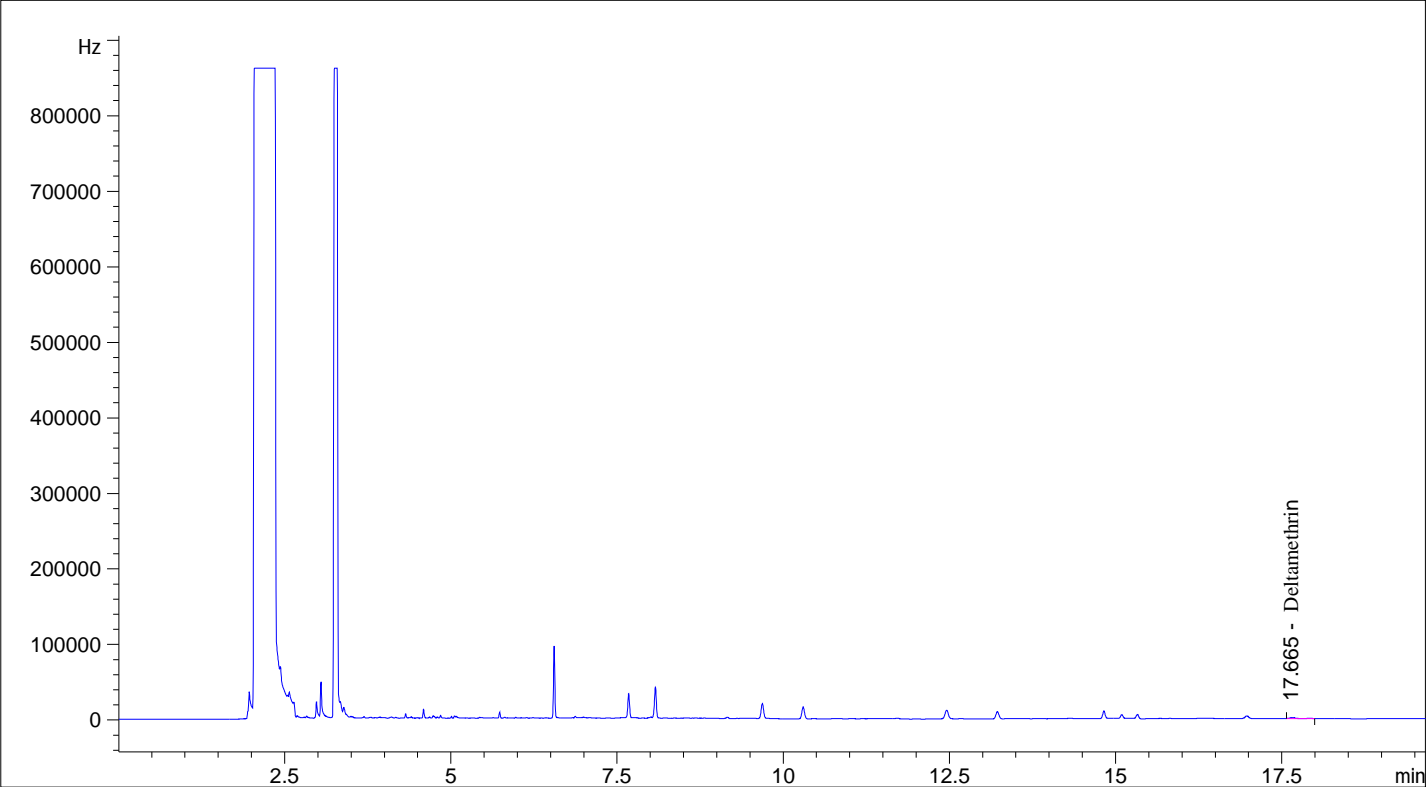

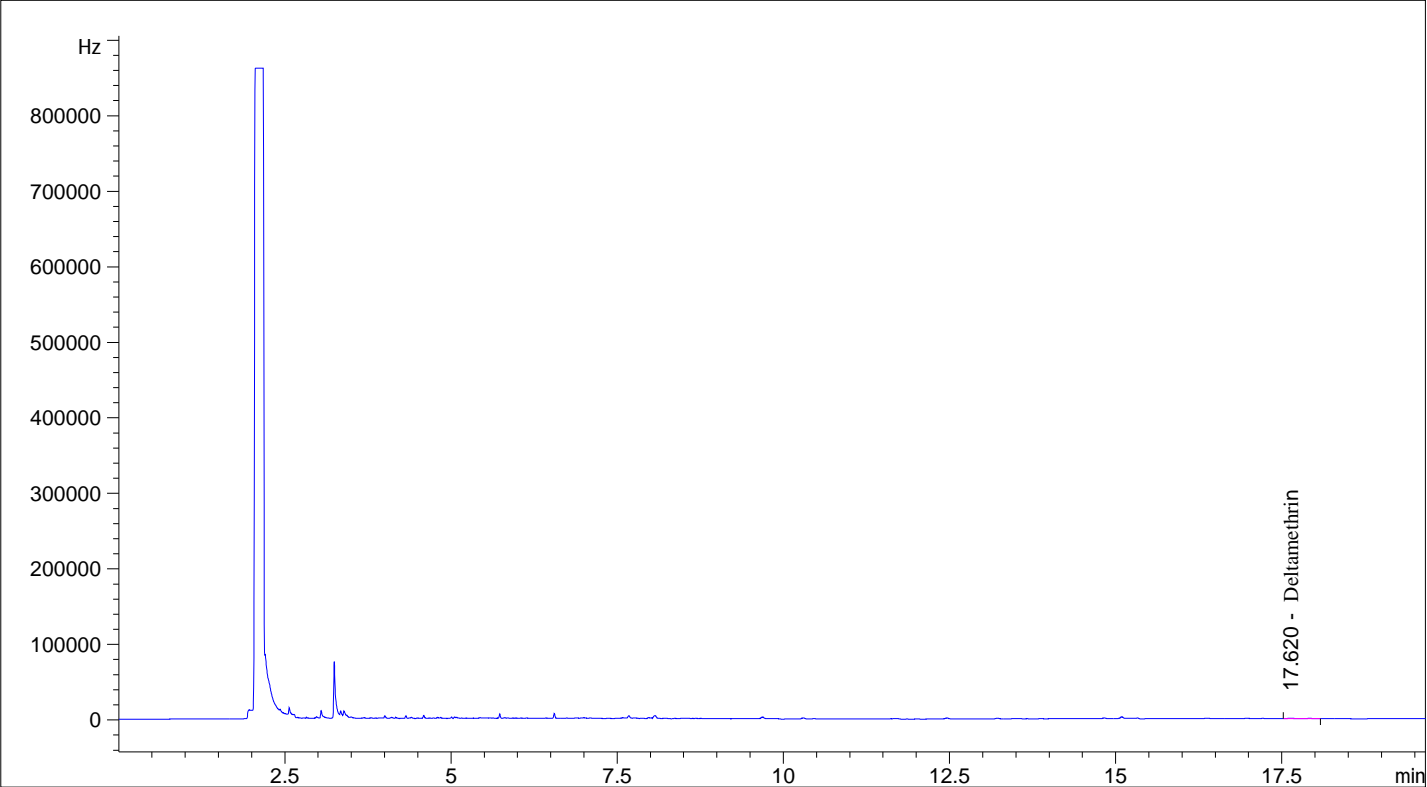

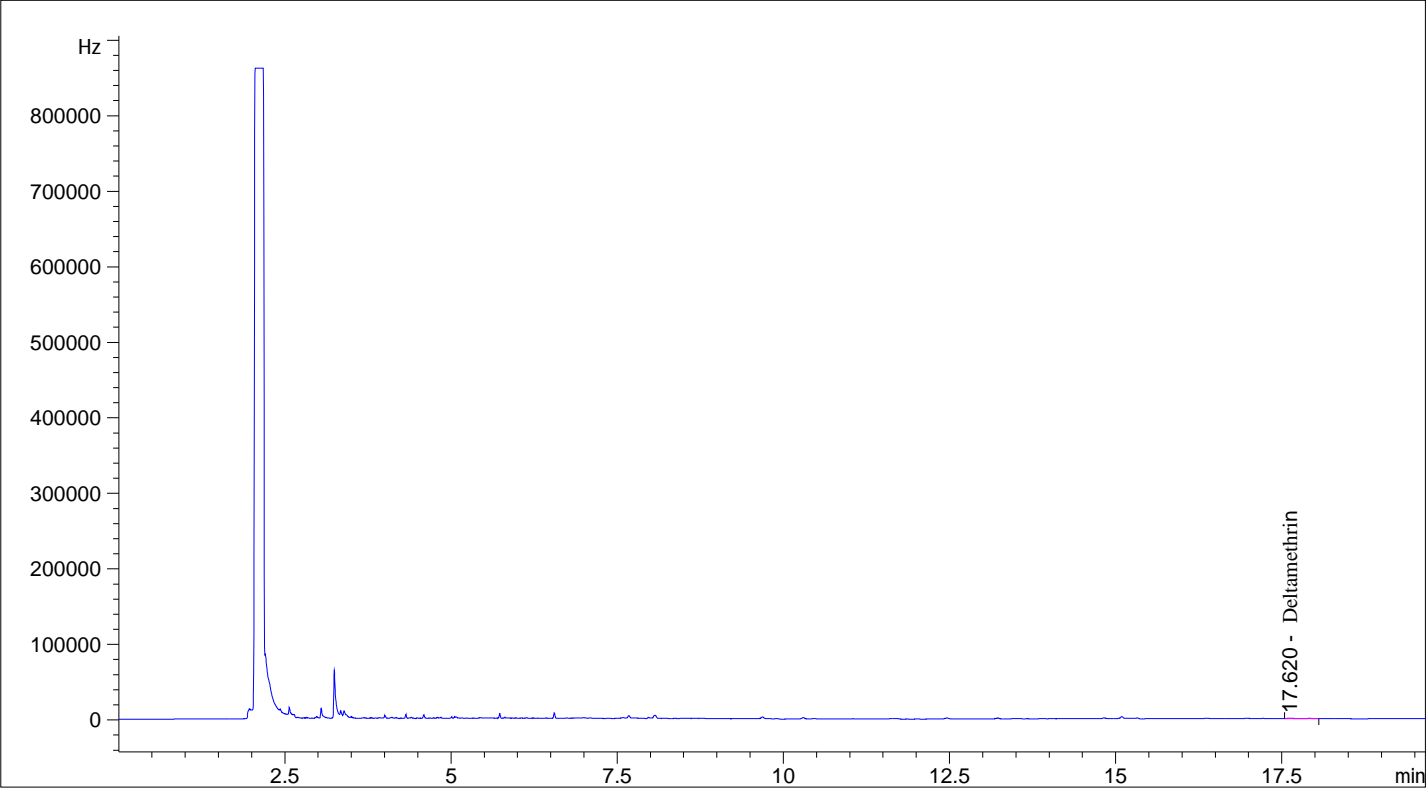

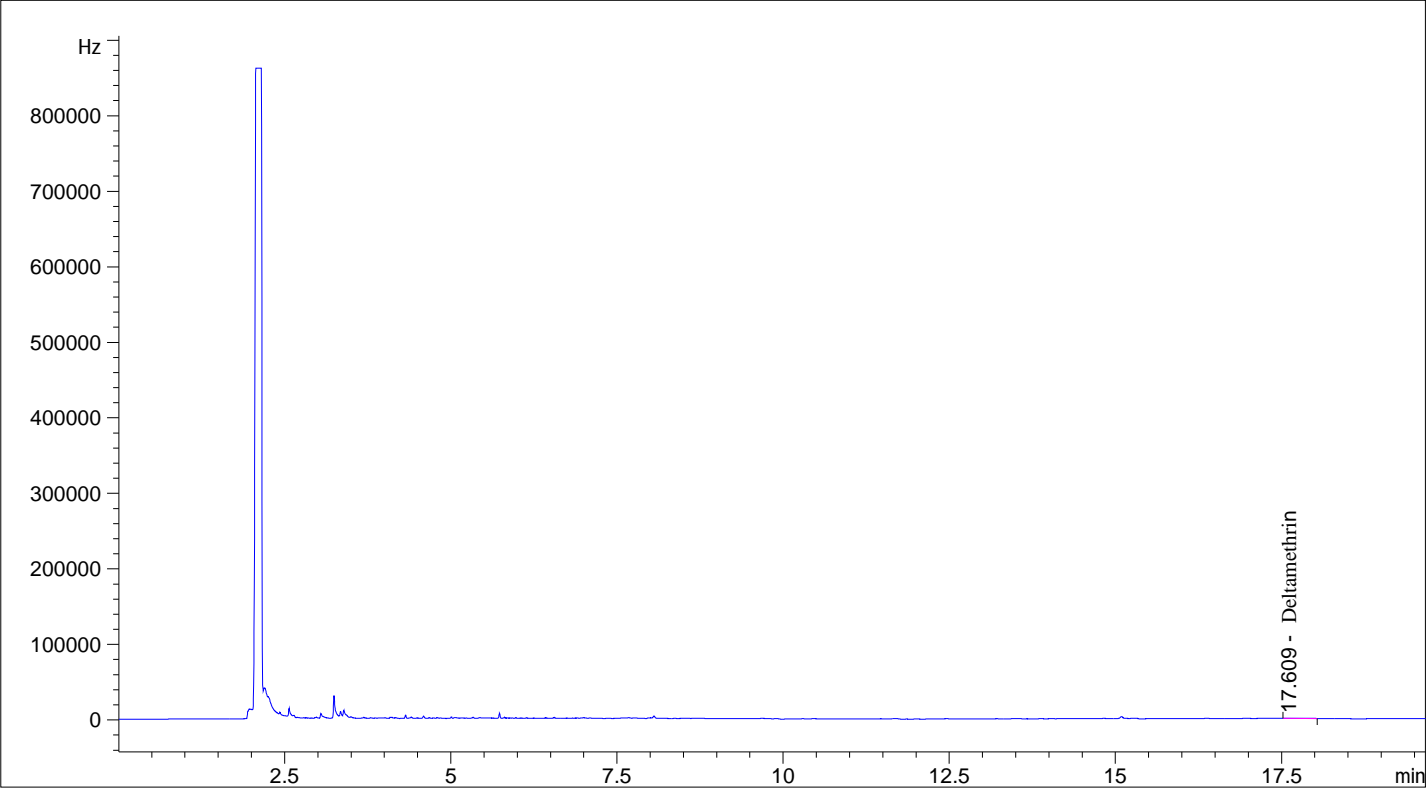

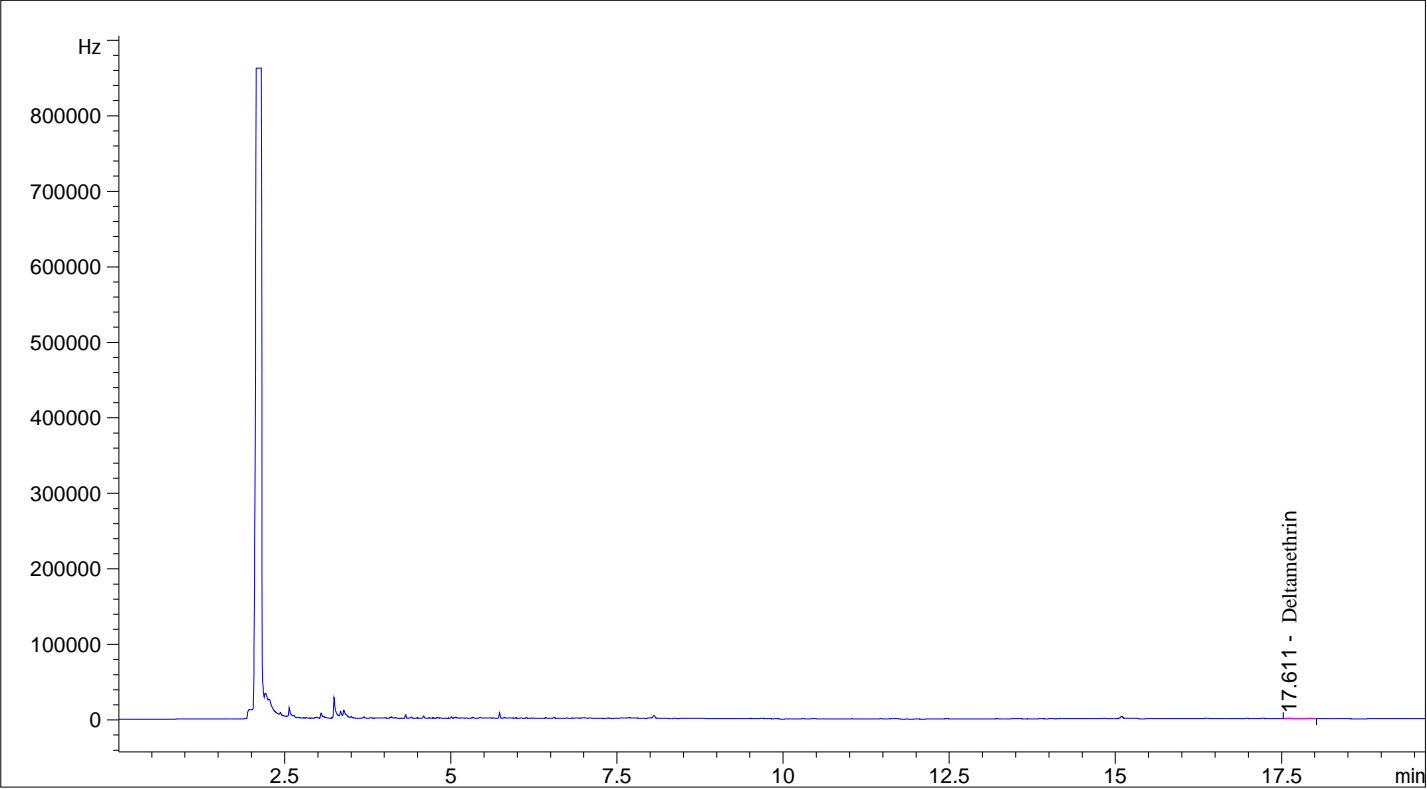

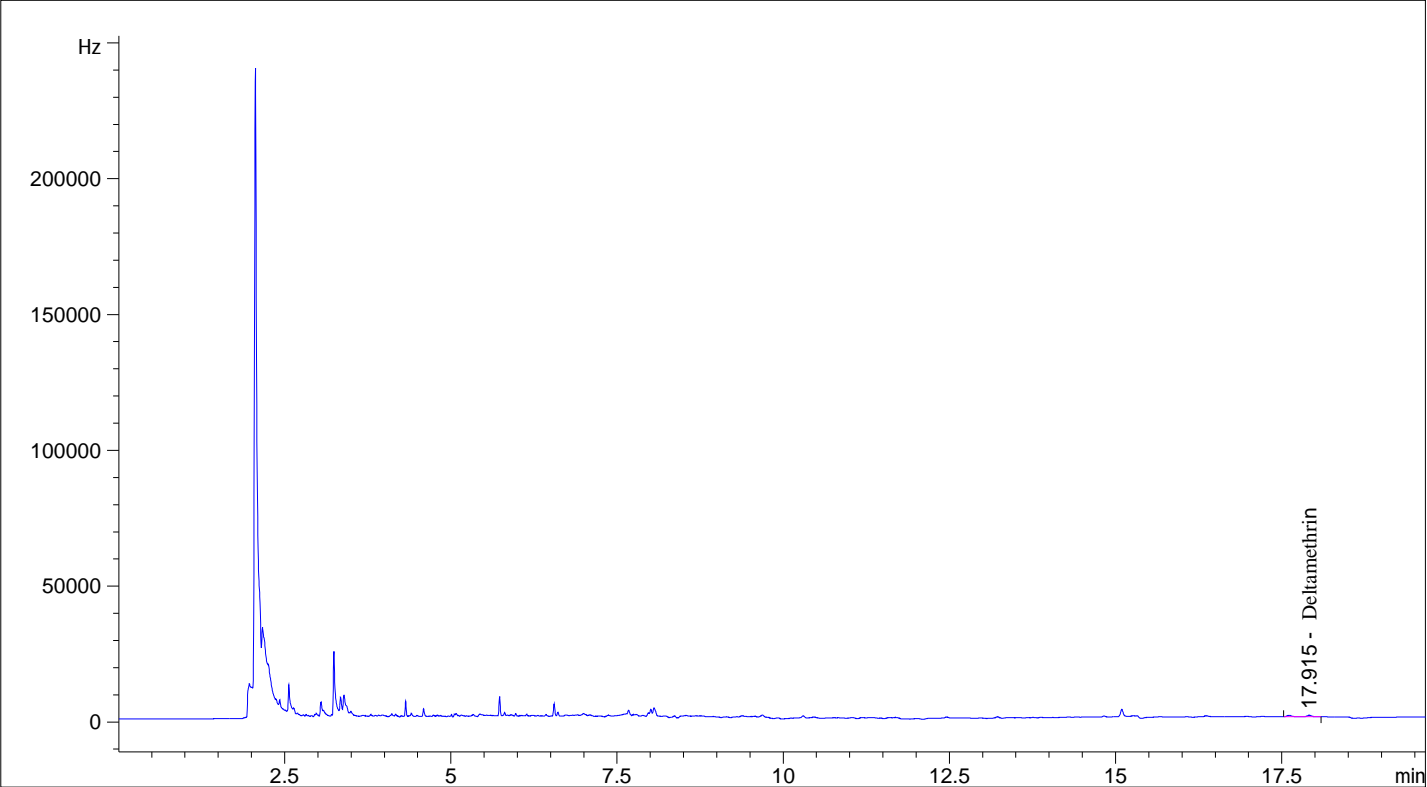

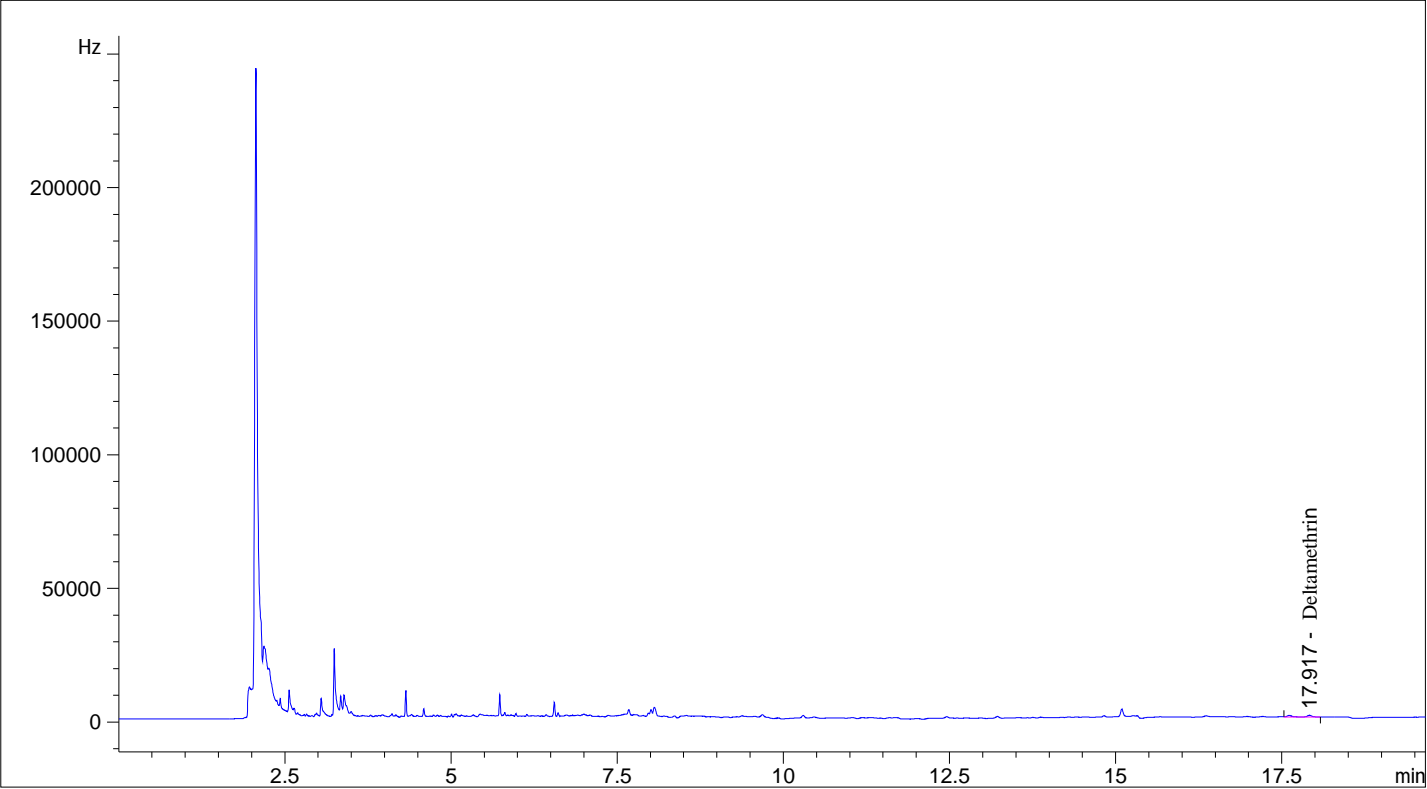

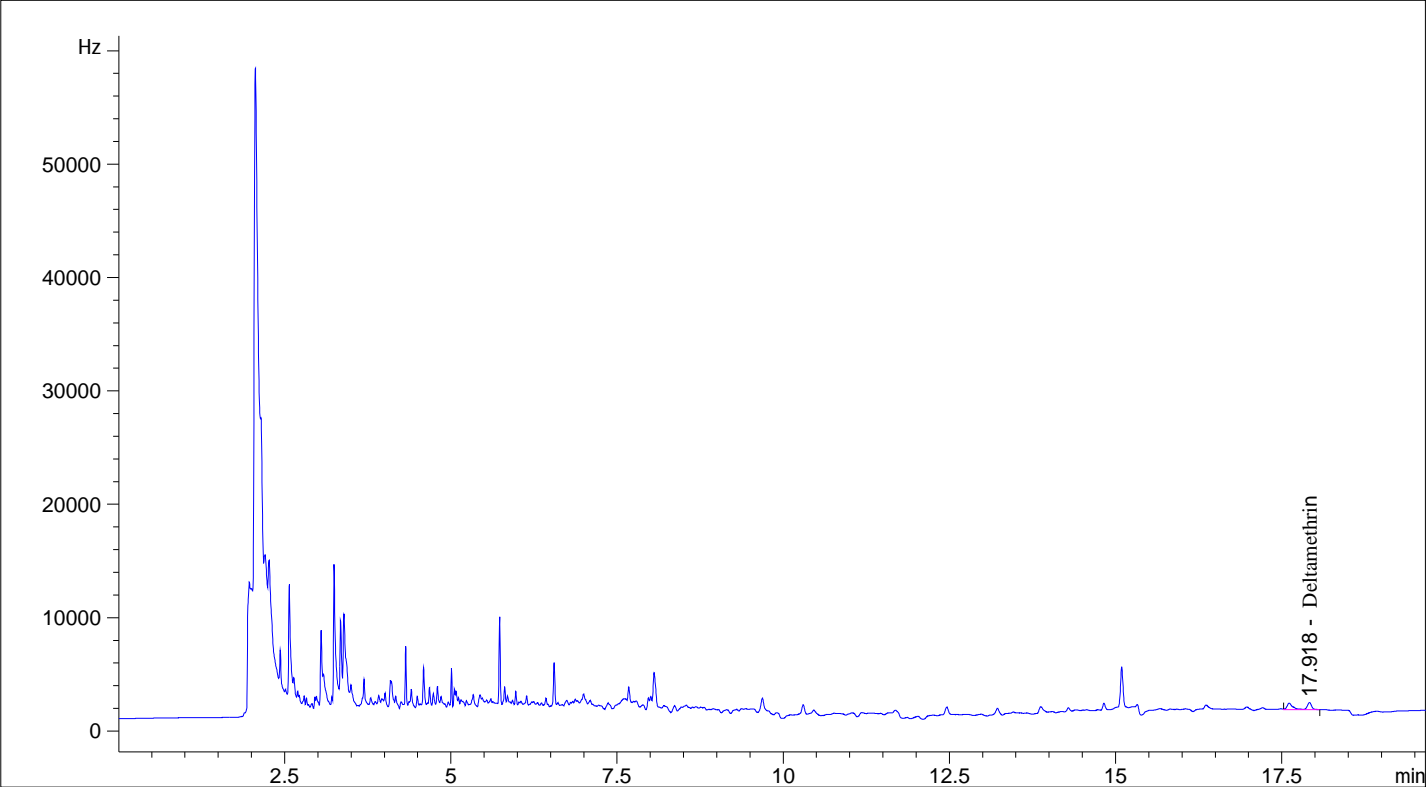

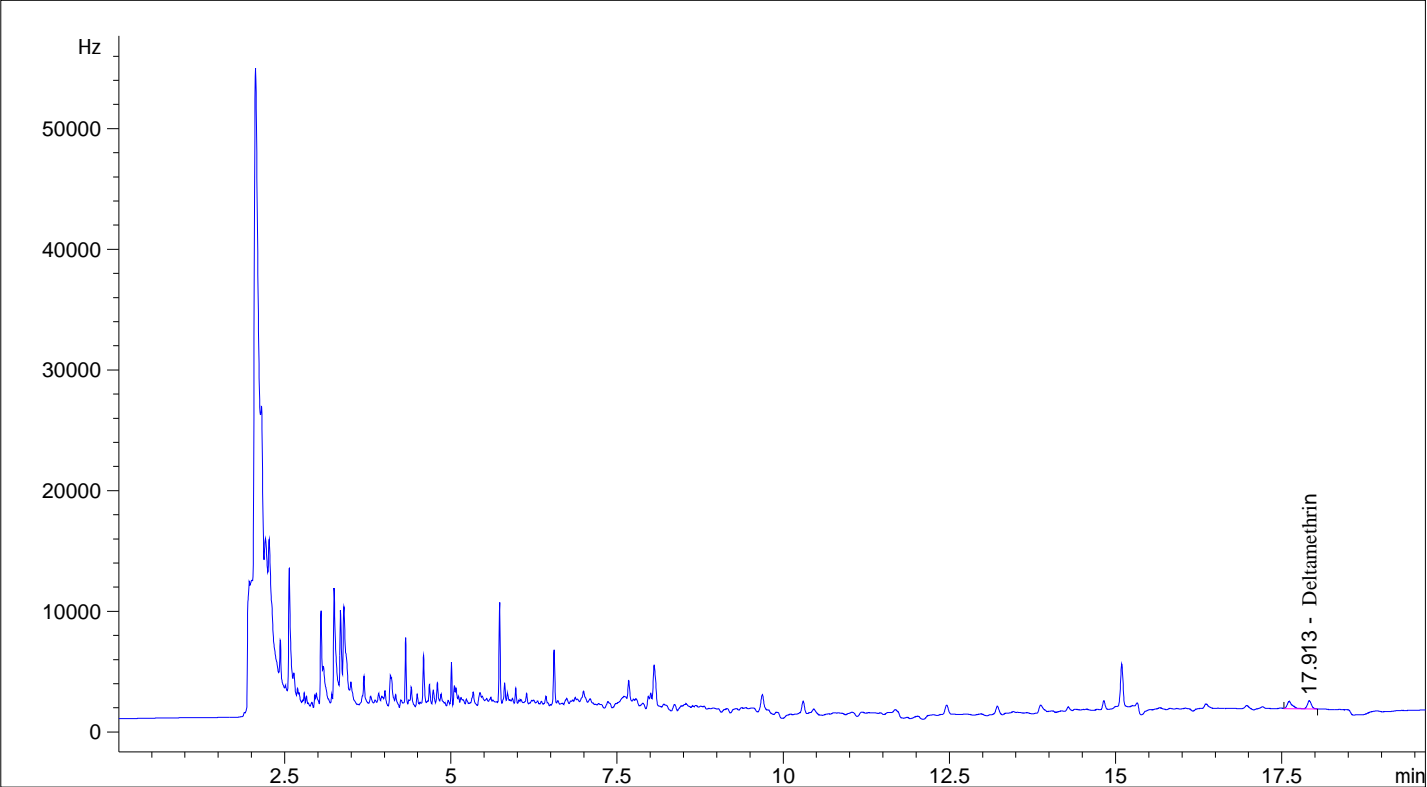

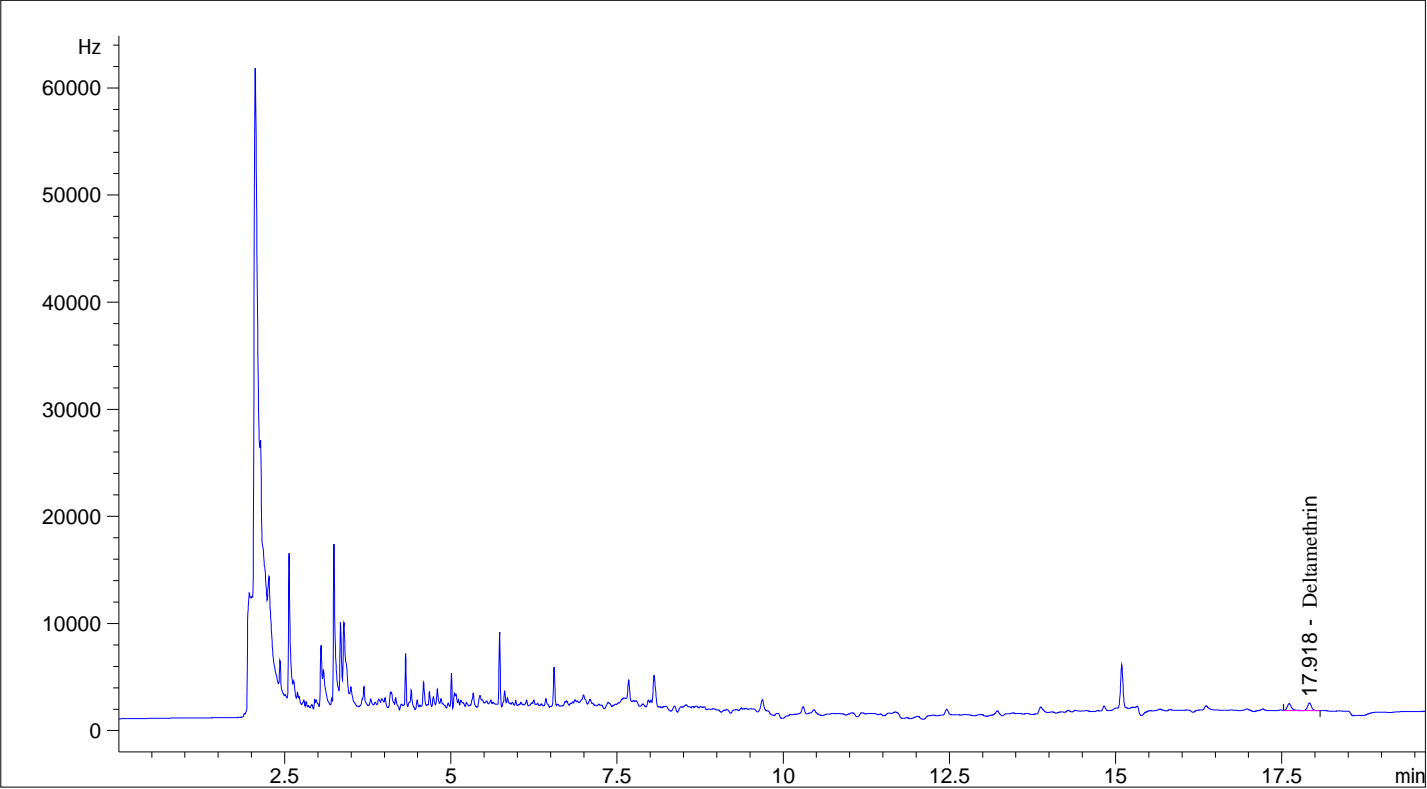

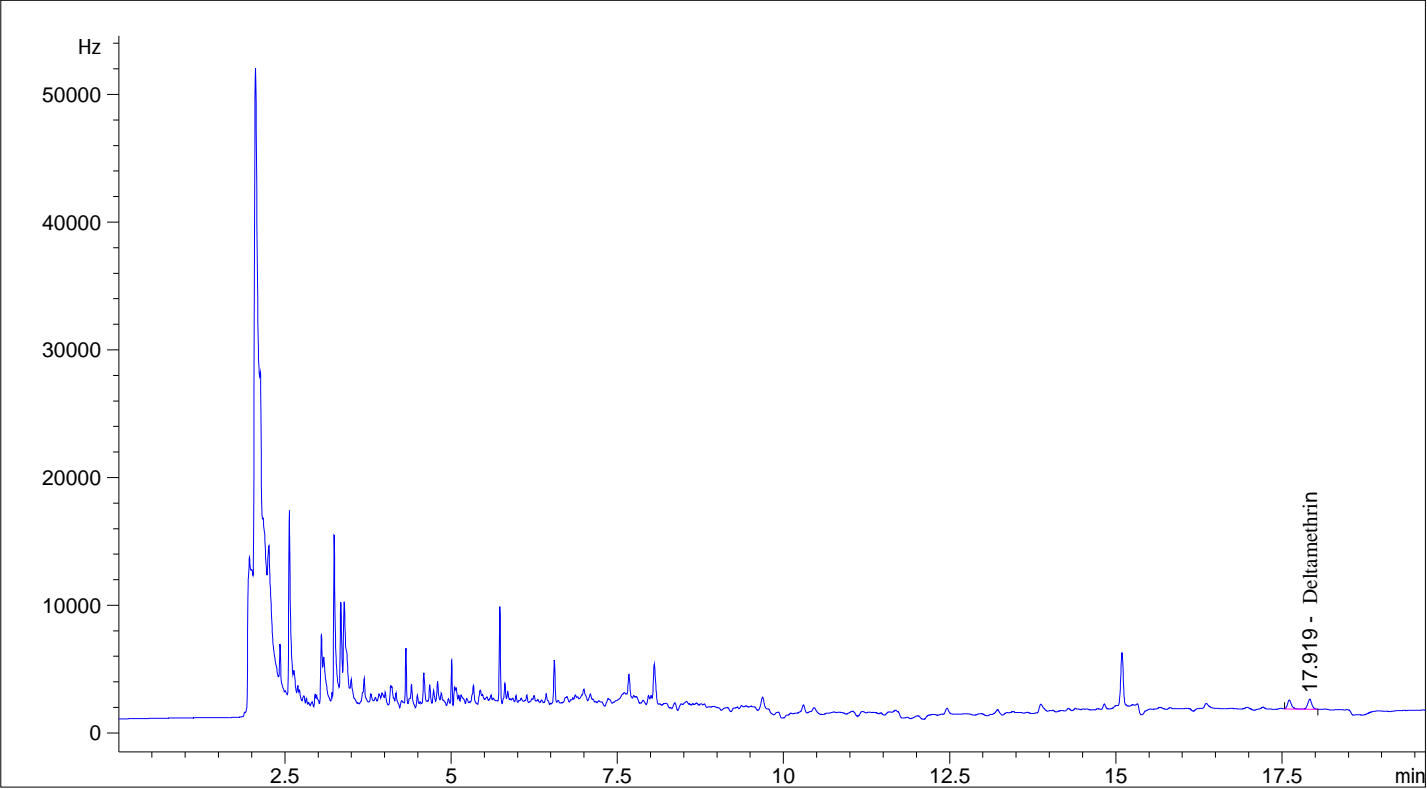

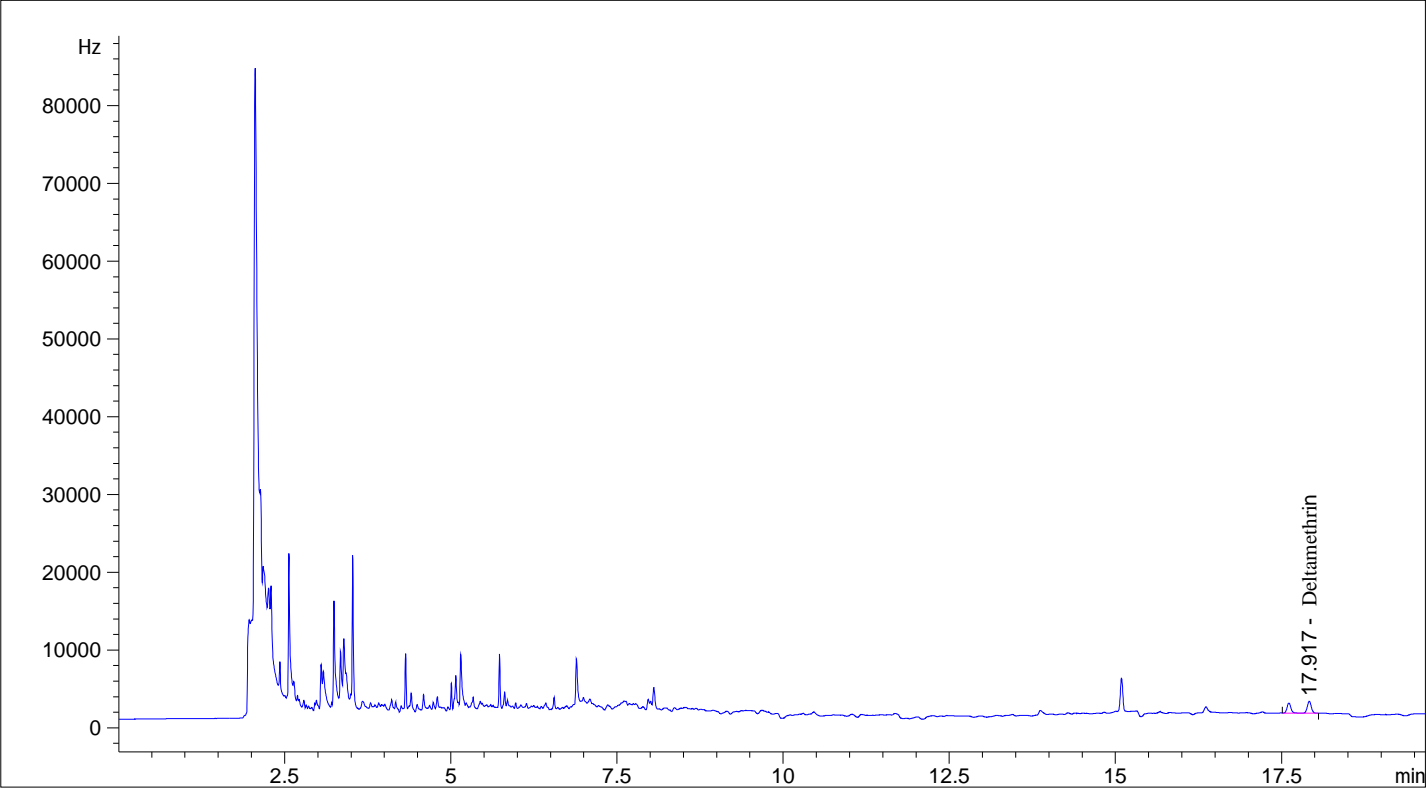

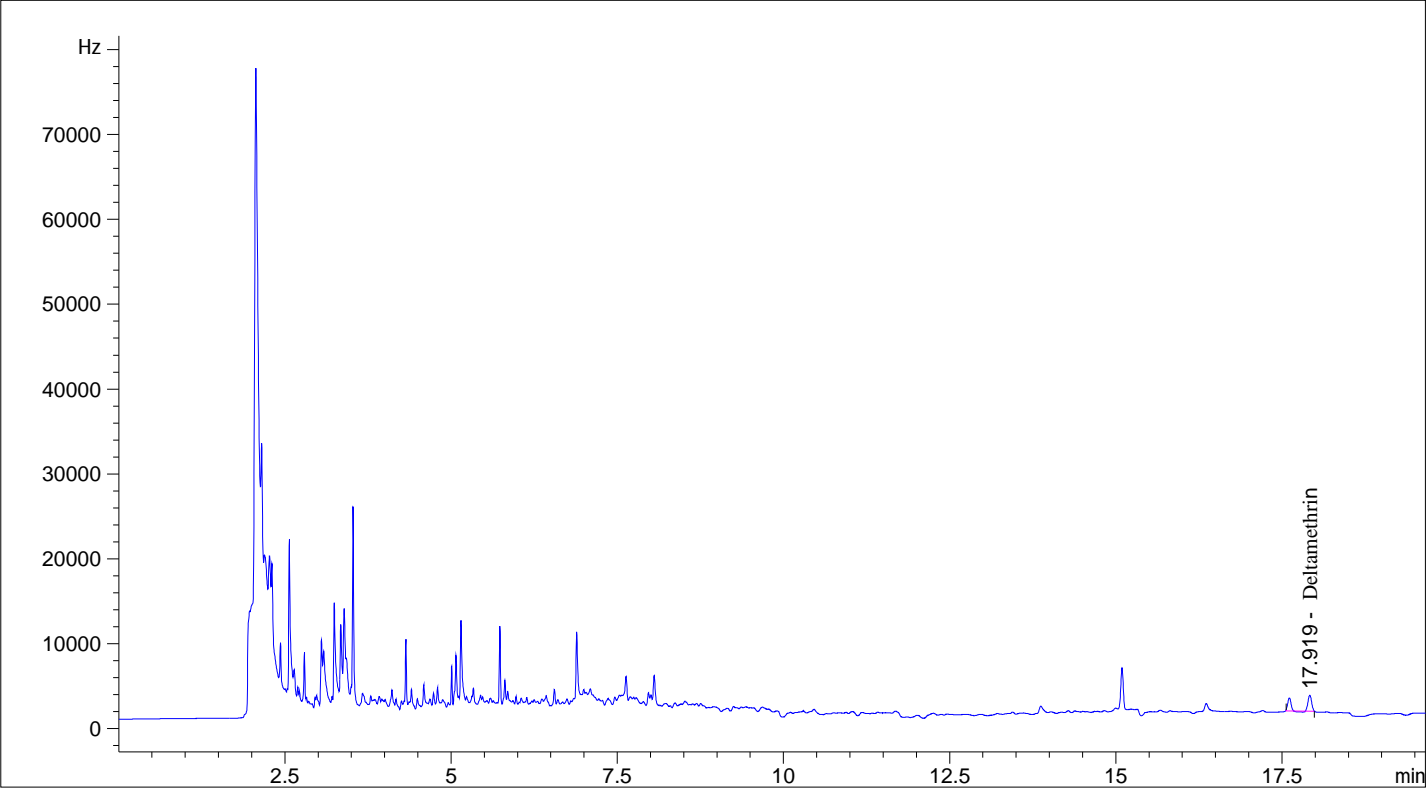

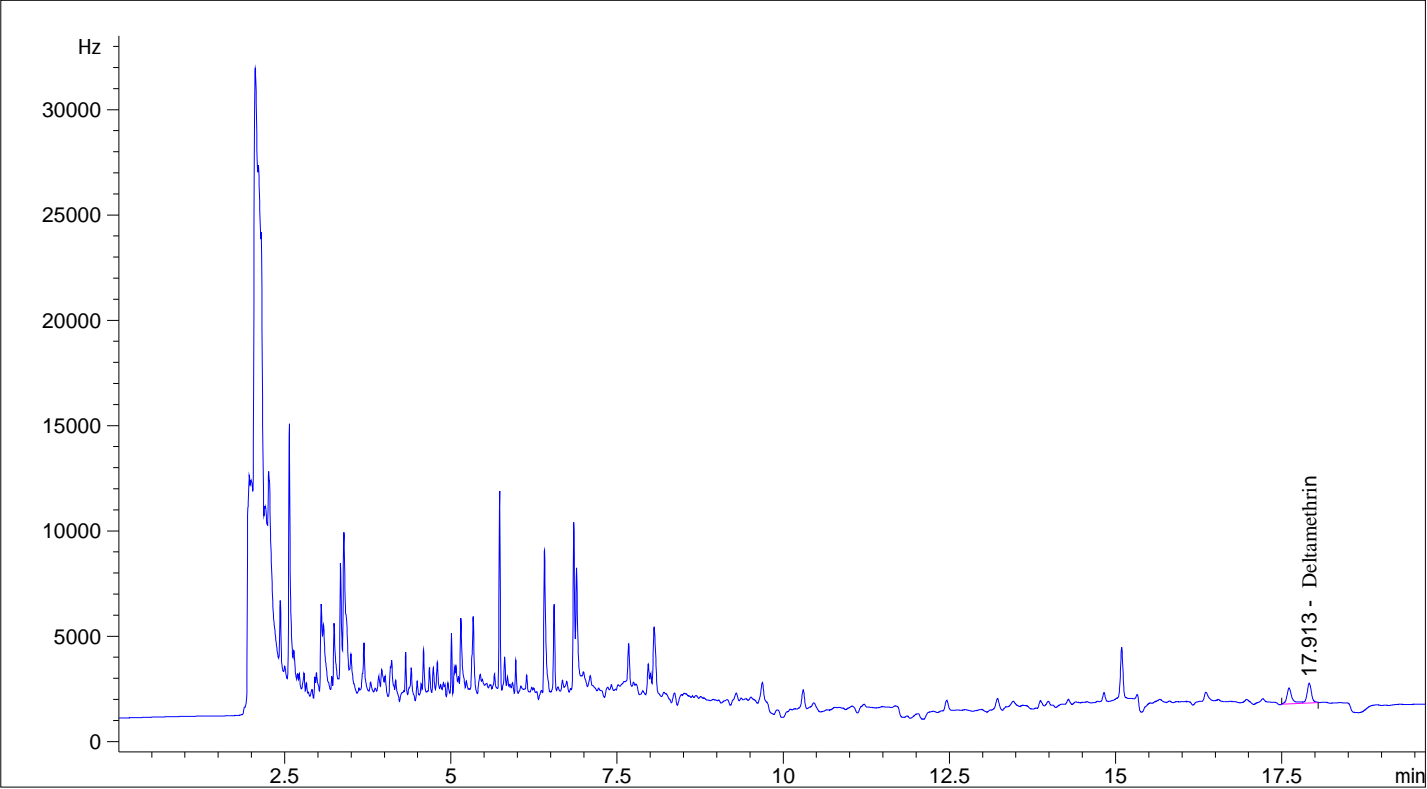

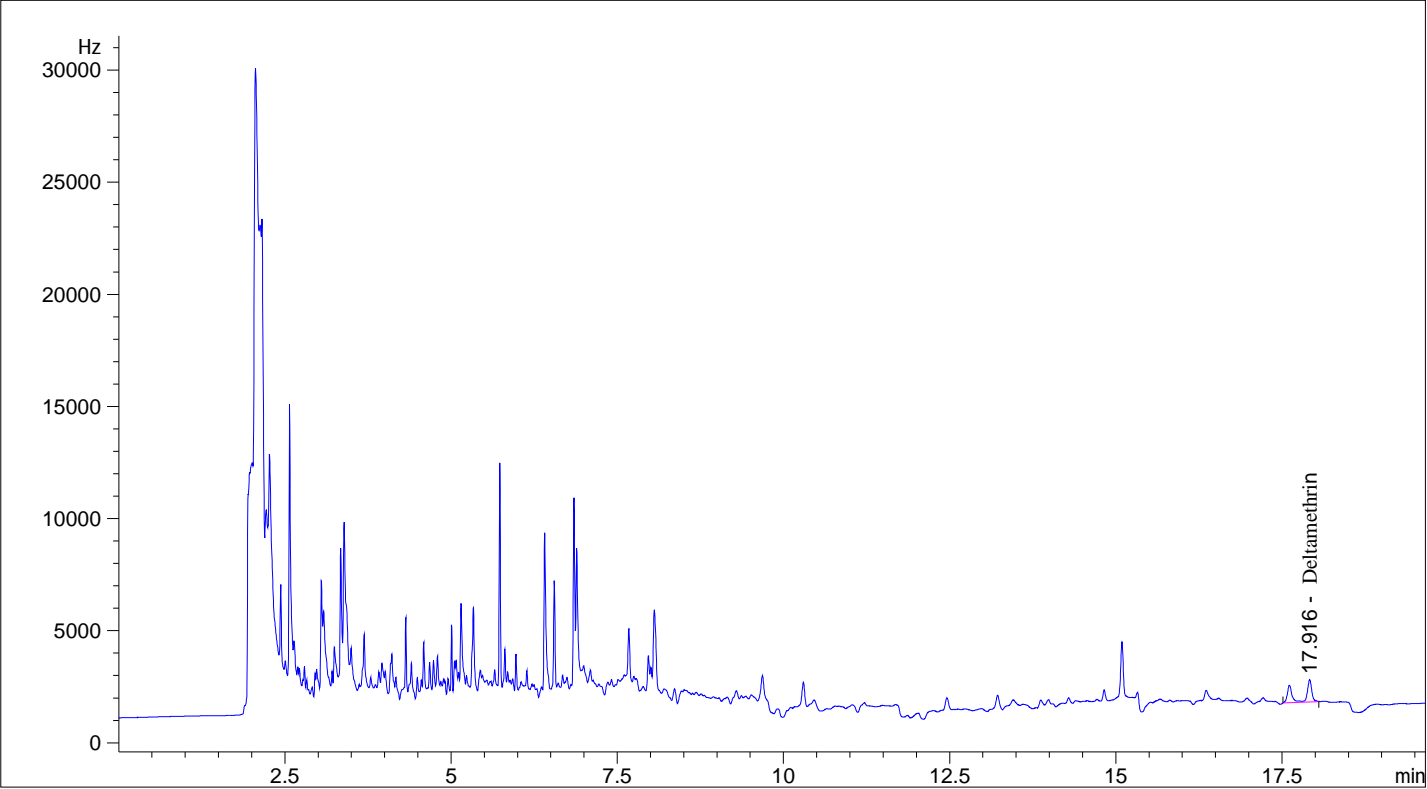

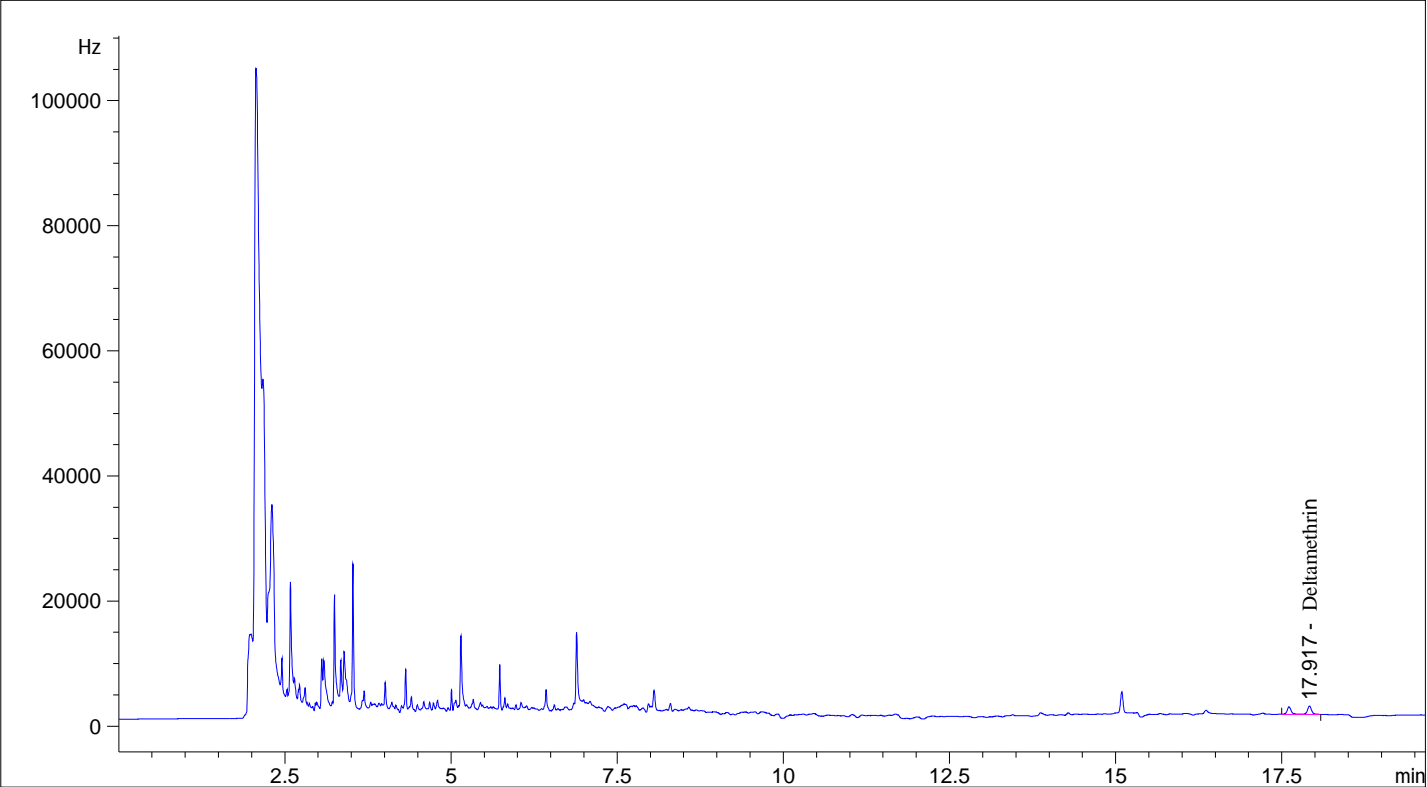

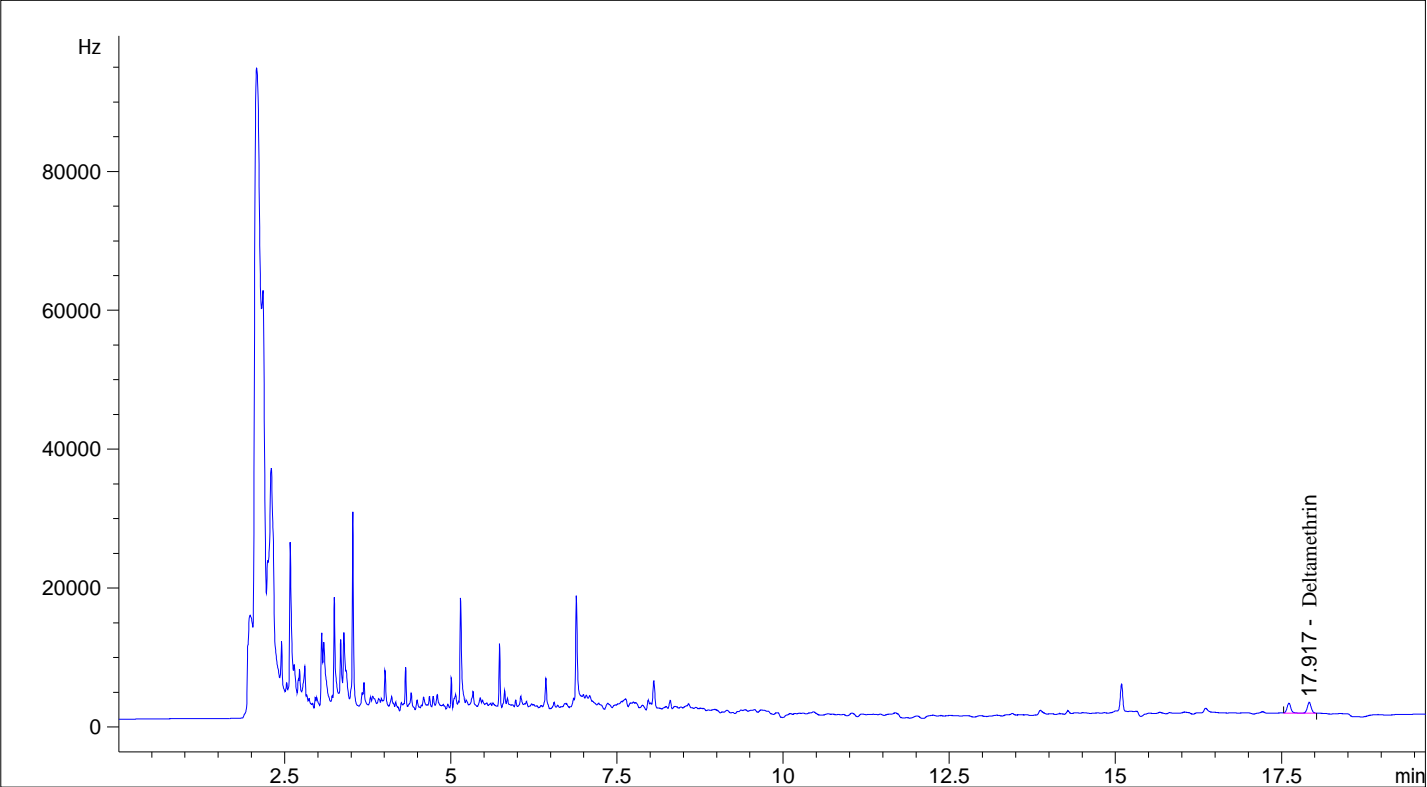

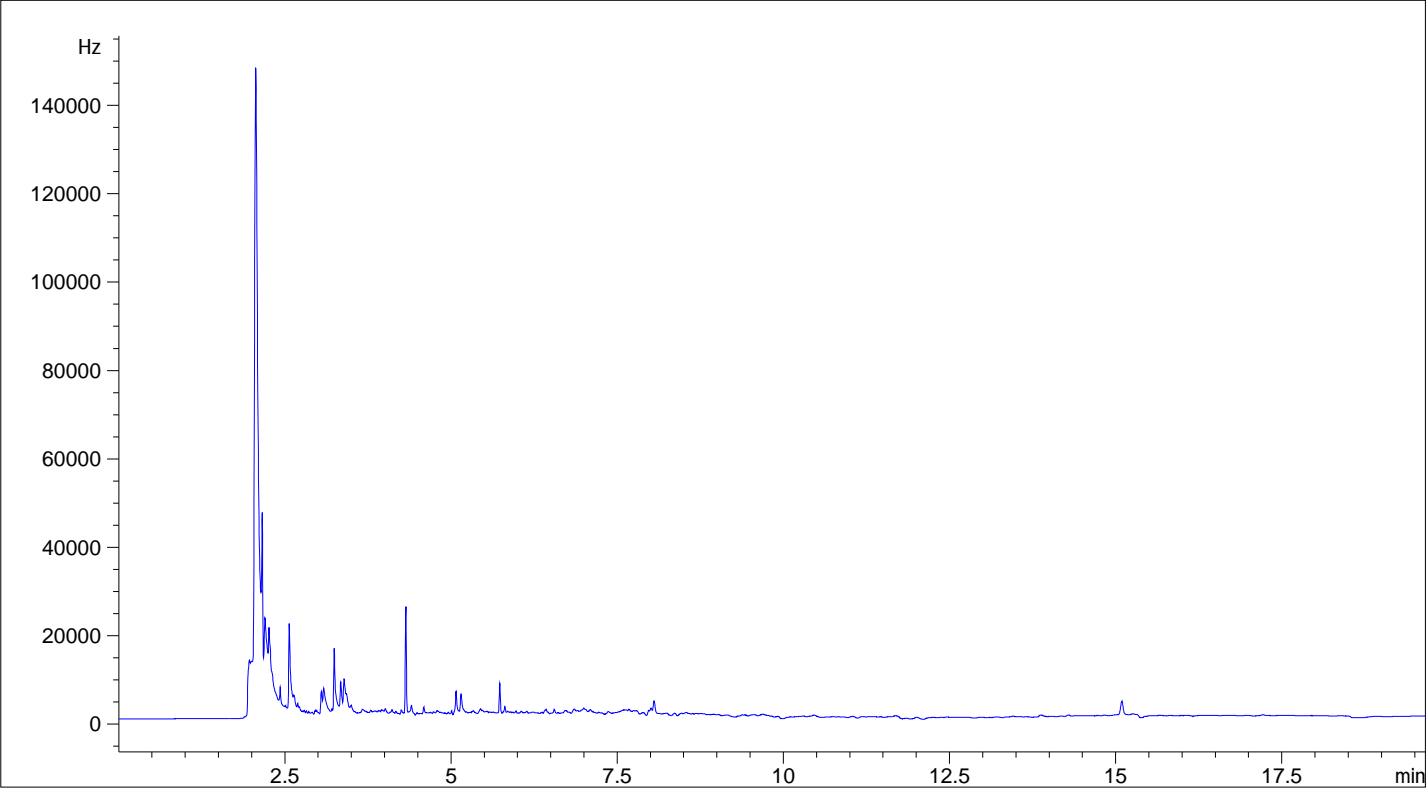

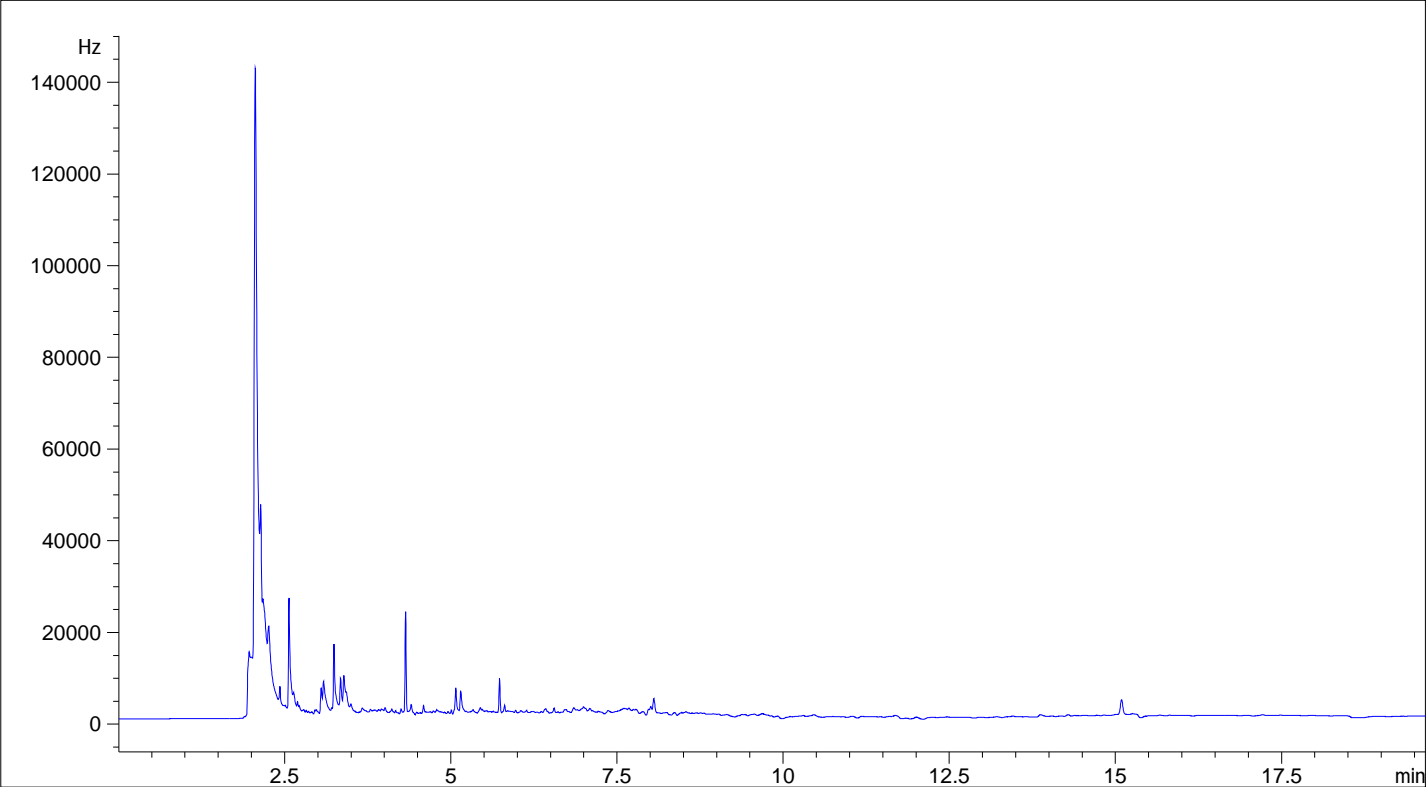

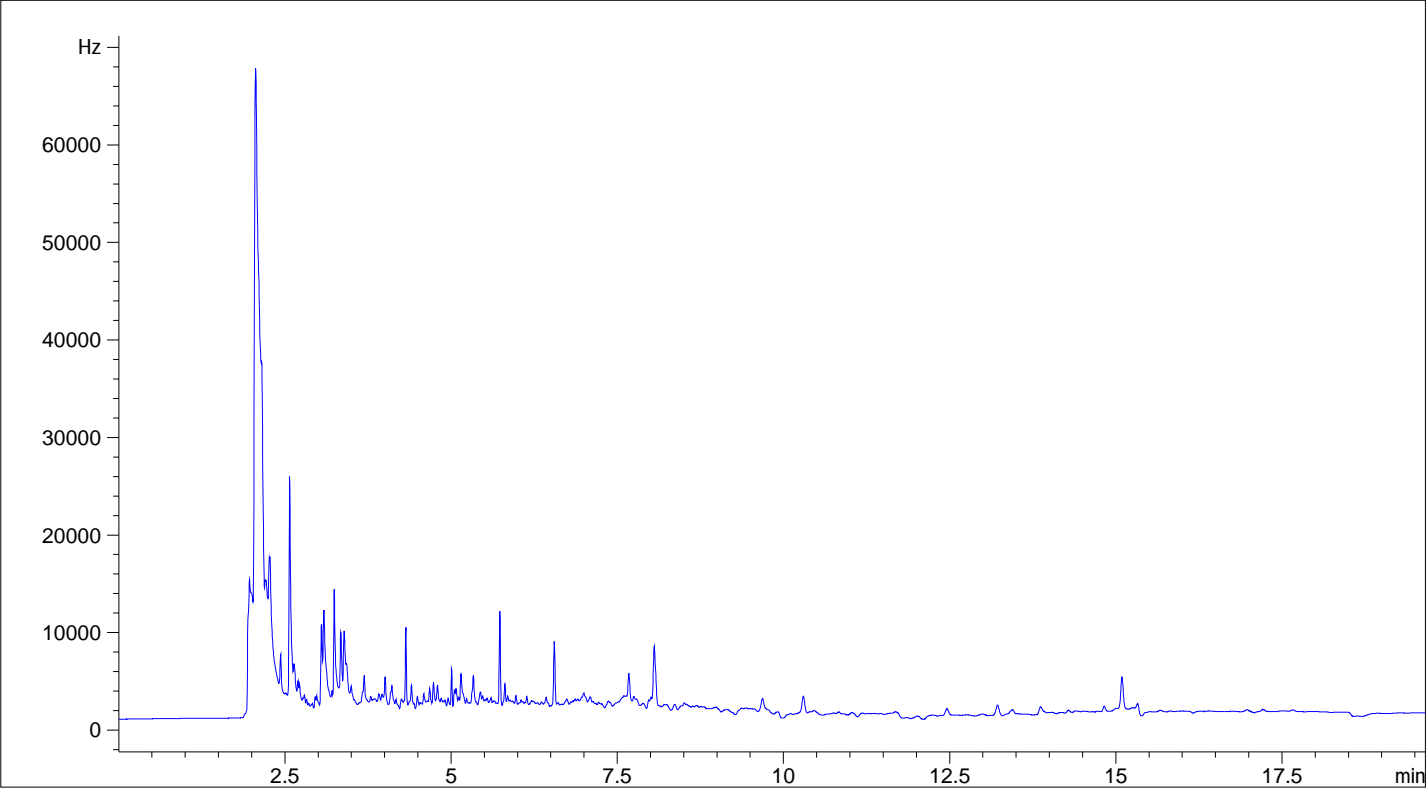

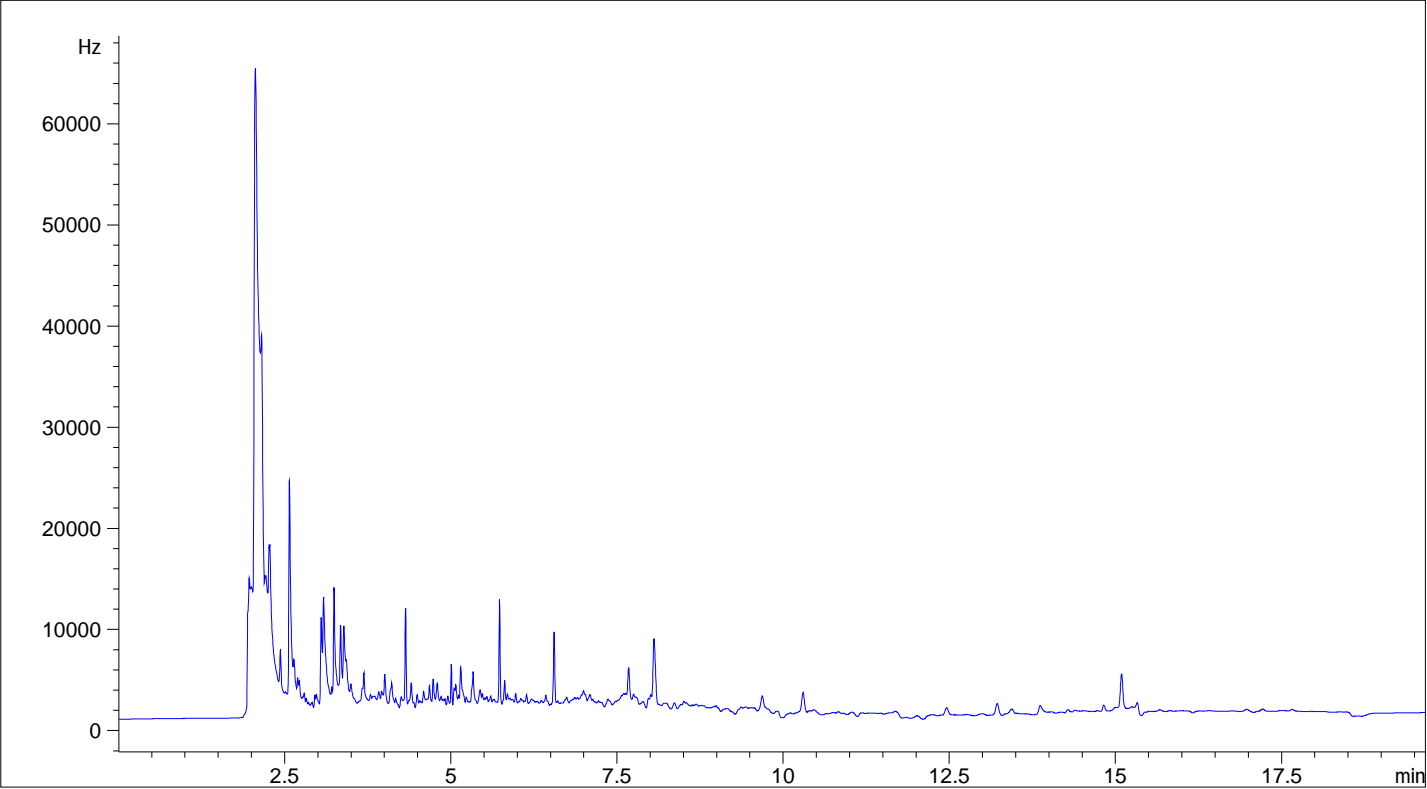

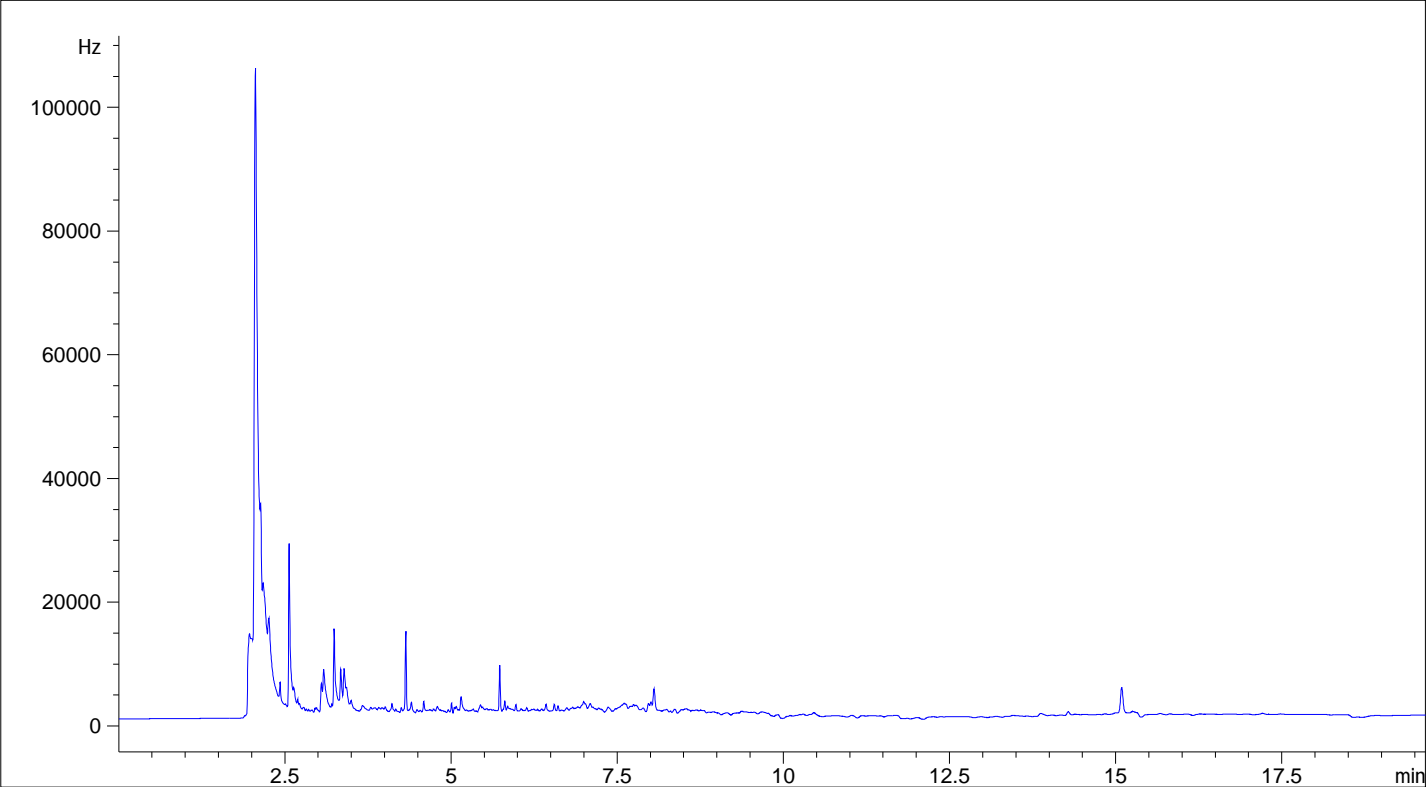

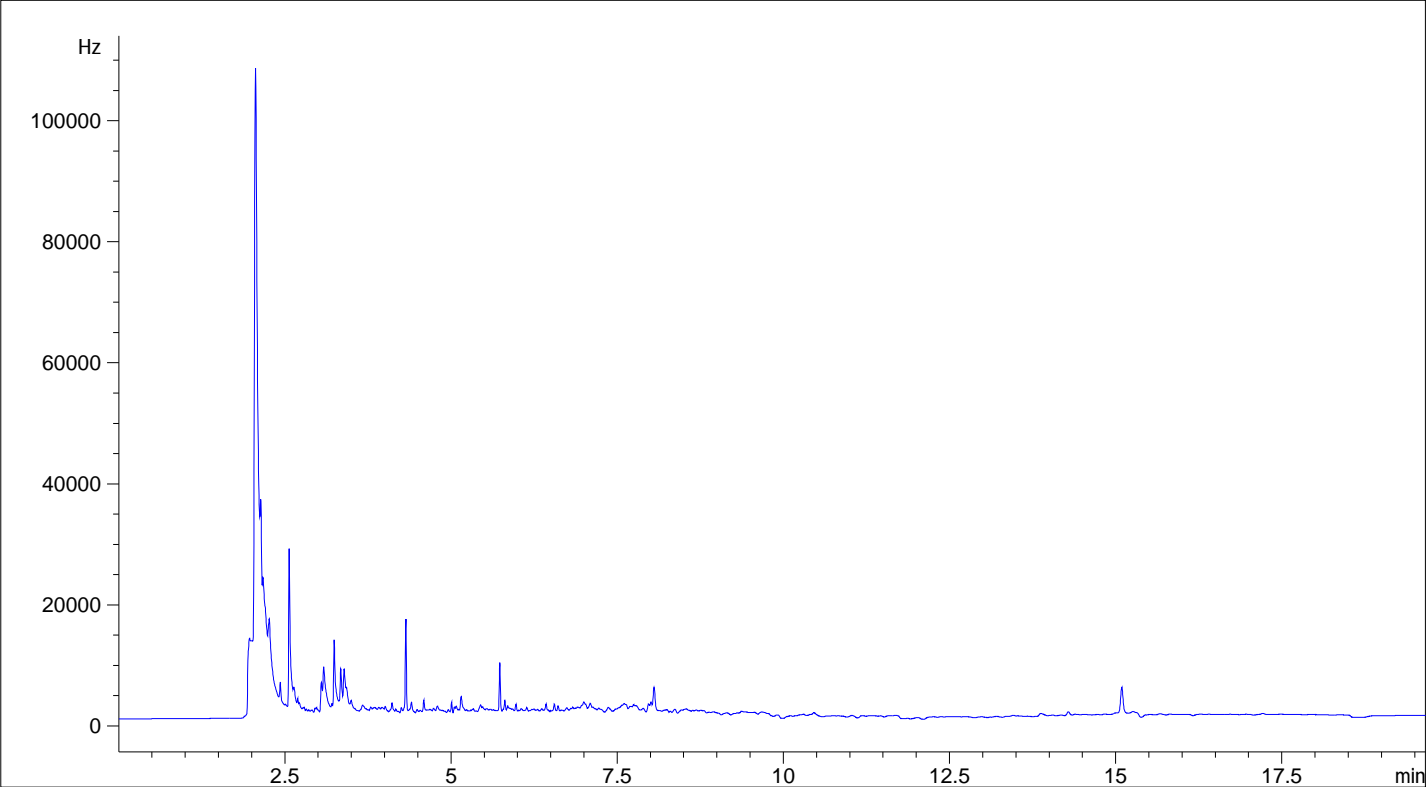

Supplement: Supplementary file 4 [file DataSheet3.pdf]

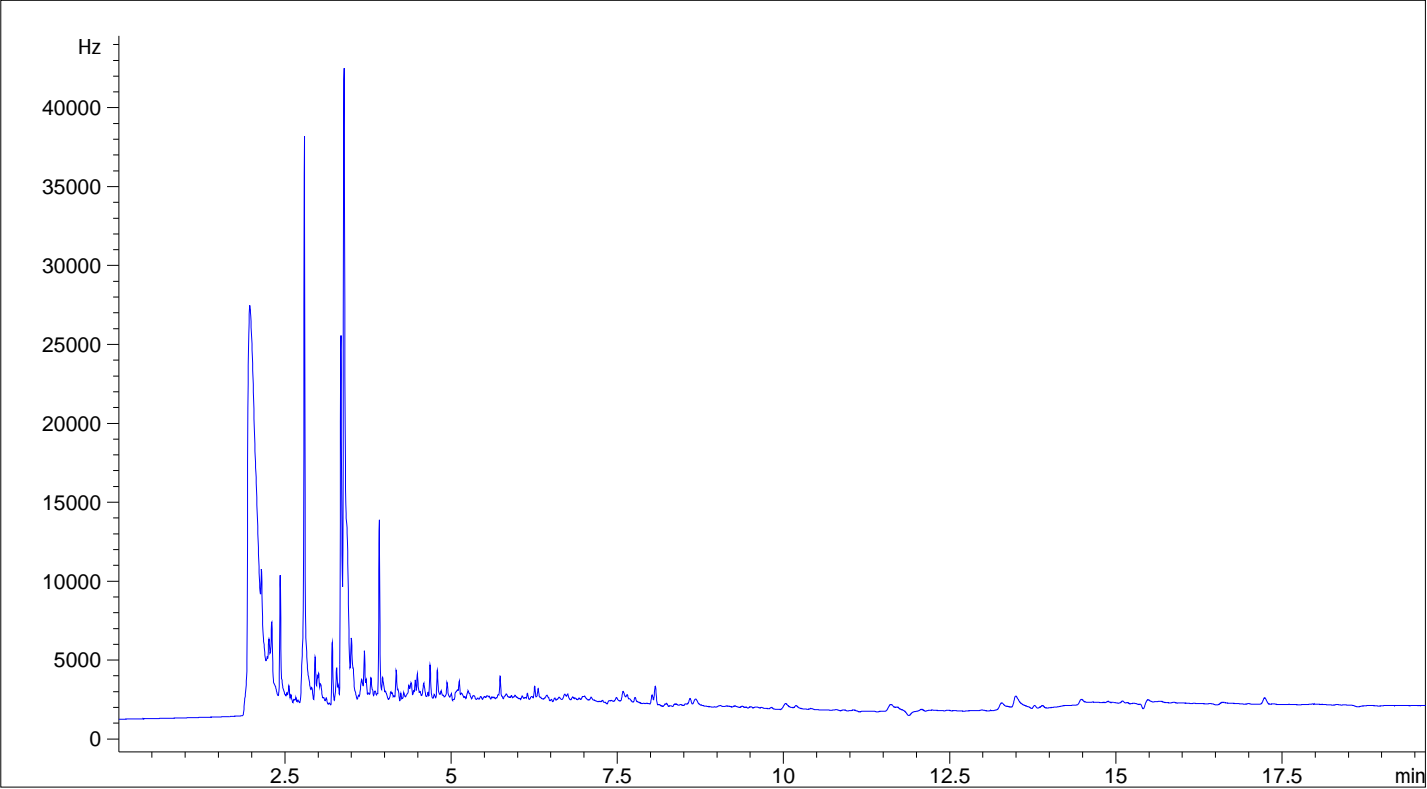

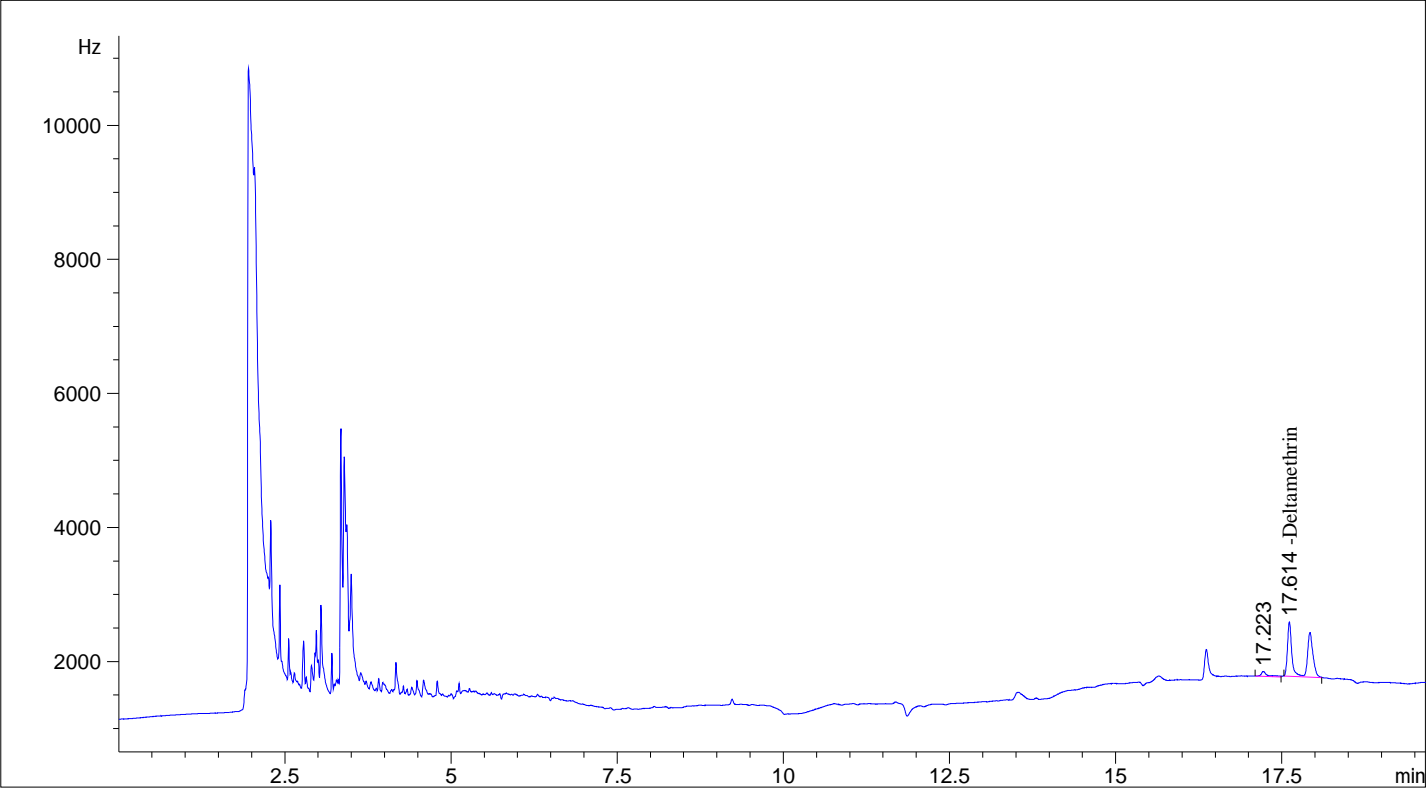

Supplement: Supplementary file 5 [file DataSheet1.pdf]
